# *N*-methyl Benzimidazole Tethered Cholic Acid Amphiphiles Can Eradicate *S. aureus*-Mediated Biofilms and Wound Infections

**DOI:** 10.3390/molecules27113501

**Published:** 2022-05-30

**Authors:** Himanshu Kakkar, Nalini Chaudhary, Devashish Mehta, Varsha Saini, Shallu Maheshwari, Jitender Singh, Preeti Walia, Avinash Bajaj

**Affiliations:** 1 Lord Shiva College of Pharmacy, Near Civil Hospital, Sirsa 125055, Haryana, India; himanshukakkar1670@gmail.com (H.K.); shallumaheshwari180@gmail.com (S.M.); saggujittu@gmail.com (J.S.); 2 Laboratory of Nanotechnology and Chemical Biology, Regional Centre for Biotechnology, NCR Biotech Science Cluster, 3rd Milestone, Faridabad-Gurgaon Expressway, Faridabad 121001, Haryana, India; nalinichaudhary2311@gmail.com (N.C.); devashish.mehta@rcb.res.in (D.M.); varsha.phd19@rcb.res.in (V.S.)

**Keywords:** *S. aureus*, membrane targeting amphiphiles, cholic acid, antimicrobial resistance

## Abstract

Infections associated with Gram-positive bacteria like *S. aureus* pose a major threat as these bacteria can develop resistance and thereby limit the applications of antibiotics. Therefore, there is a need for new antibacterials to mitigate these infections. Bacterial membranes present an attractive therapeutic target as these membranes are anionic in nature and have a low chance of developing modifications in their physicochemical features. Antimicrobial peptides (AMPs) can disrupt the microbial membranes via electrostatic interactions, but the poor stability of AMPs halts their clinical translation. Here, we present the synthesis of eight *N*-methyl benzimidazole substituted cholic acid amphiphiles as antibacterial agents. We screened these novel heterocyclic cholic acid amphiphiles against different pathogens. Among the series, CABI-**6** outperformed the other amphiphiles in terms of bactericidal activity against *S. aureus.* The membrane disruptive property of CABI-**6** using a fluorescence-based assay has also been investigated, and it was inferred that CABI-**6** can enhance the production of reactive oxygen species. We further demonstrated that CABI-**6** can clear the pre-formed biofilms and can mitigate wound infection in murine models.

## 1. Introduction

*S. aureus* is an opportunistic Gram-positive pathogen that causes lethal infections like sepsis, endocarditis, and pneumonia. *S. aureus* can also cause intracellular infections by evading phagocytosis [1]. Currently available antimicrobial drugs are rendered ineffective after a certain period of their launch as bacteria demonstrate the ability to evolve resistance. Since commonly used therapies target important components of bacterial cells’ metabolic and cell wall machinery, these targets are prone to genetic mutations and are responsible for drug resistance [2]. Bacteria gain resistance against antimicrobial agents via different mechanisms including the formation of biofilms, the modification of drug targets, the development of efflux pumps, and the secretion of drug-degrading enzymes [3,4]. Recently, antimicrobial resistance (AMR) and the increased growth rate of bacterial infections have resulted in high mortality rates, and the development of new antimicrobial therapies has been drastically reduced. Therefore, there is an urgent necessity to develop novel antimicrobial agents to target bacterial infections without allowing them to gain resistance [5].

Membranes of Gram-positive bacterial cells present a unique biochemical composition with teichoic acid (TA) as a structural component. TA is composed of glycerol/ribitol phosphate and carbohydrate moieties connected via phosphodiester linkages and is covalently bound to peptidoglycan [6,7]. The phosphate-rich components in TA contribute to the negative charge of bacterial cell membranes [8]. The disruption of the bacterial membrane is both a selective and effective approach to tackling antimicrobial resistance that can be used for next-generation antimicrobial therapy [9]. Antimicrobial peptides (AMPs) are part of the innate immune system and possess potent antimicrobial properties against Gram-positive and Gram-negative pathogens [10]. Mechanistically, AMPs disrupt the bacterial membrane via electrostatic interactions as they are cationic in nature. However, the stability of AMPs limits their clinical usage against bacterial infections. Therefore, the engineering of membrane-targeting molecules could be a beneficial therapeutic approach to overcoming AMR [11].

Cholic acid (CA) is a primary bile acid that is synthesized via cholesterol metabolism, and it plays a crucial role in lipid metabolism [12]. The presence of both a steroidal backbone and hydroxyl groups provide a facial amphiphilic nature to CA and also the potential to behave as mimics of AMPs [13]. Derivatives of CA have been widely exploited as antimicrobial [14], anticancer [15], and diagnostic agents [16]. Savage and coworkers in their pioneering work developed bile acid-based antimicrobial agents (called Ceragenins) that demonstrated broad-spectrum antibacterial properties against different Gram-positive and Gram-negative pathogens [17,18,19,20]. Rahman et al. reported bile acid-derived polymeric compounds as effective antibacterial agents that can disrupt the Gram-negative bacterial membrane with negligible cytotoxicity [21]. Recently, Salunke’s group synthesized dimer, trimer, and tetramers of cholic and deoxycholic acid, and showed that dimeric compounds possess an antibiotic adjuvant property that leads to a reduction in the minimum inhibitory values of antibiotics [22].

**Figure 1:**
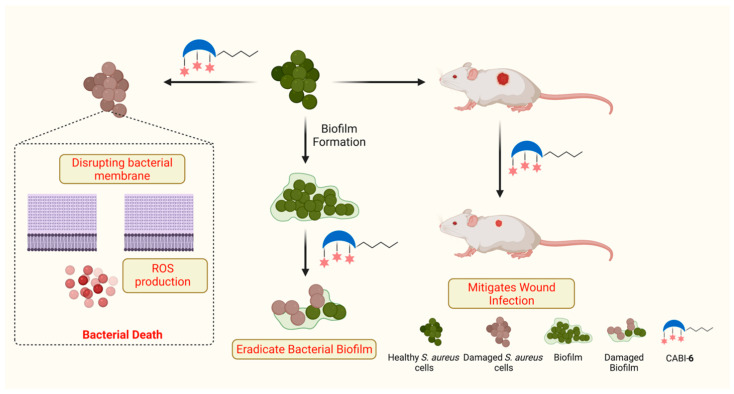
Schema illustrating the antibacterial activities of synthesized amphiphile.

Benzimidazole is a fused heterocyclic functional moiety that contains a benzene ring fused with an imidazole group, and benzimidazole derived molecules have displayed numerous pharmacological properties including anticancer [23], antimicrobial [24], and anti-inflammatory properties [25]. Researchers have demonstrated that derivatives of benzimidazole can act as antibacterial agents. Zhang et al. synthesized a series of quinolone-benzimidazoles hybrid molecules and demonstrated their membrane-disrupting features against Gram-positive and Gram-negative pathogens [26]. Recently, Dokla’s group developed benzimidazole-based antimicrobial molecules, and demonstrated that a *p*-methyl benzyl substituted molecule can unlock the bacterial membrane and allow colistin to penetrate inside the bacteria [27]. Here, we present the design and synthesis of *N*-methyl benzimidazole conjugated CA amphiphiles, and their antibacterial activities against Gram-positive and Gram-negative bacterial strains. In addition, we present the results of our further testing of the membrane-disrupting property of the most active amphiphile and its ability to clear biofilms and wound infections (Figure 1).

## 2. Results and Discussion

### 2.1. Design and Synthesis of CA-Benzimidazole Amphiphiles

Our research group has been developing bile acid-derived cationic amphiphiles and testing them for their antibacterial, antifungal, and antimycobacterial activities. We reported a series of nine CA-derived amphiphiles where three glycine moieties were tethered to the hydroxyl positions and different alkyl chains were installed on the carboxyl acid of CA [23,24,25]. Among them, hexyl conjugated amphiphiles showed potent antimicrobial activity against Gram-positive, Gram-negative, and fungal pathogens [28,29,30]. Moreover, this amphiphile can be used to control *Xoo* infections in rice plants [31]. Apart from this, we also designed and synthesized cholic acid peptide conjugates (CAPs) where twenty different natural amino acids were tethered to three hydroxyl groups of benzylated CA via glycine linker. The valine-tethered CAP was found to be an effective antibacterial agent against Gram-negative pathogens and was capable of eradicating polymicrobial biofilms [32,33,34]. Mechanistically, these molecules possess the ability disrupt the bacterial membrane leading to bacterial death.

**Figure 2:**
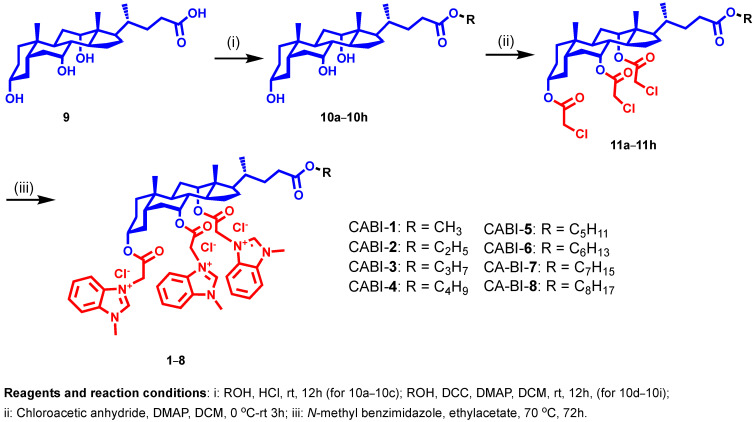
Schema illustrating the synthesis of *N*-methyl benzimidazole cholic acid amphiphiles.

In our continuing efforts to develop membrane-targeting antibacterial agents, we selected *N*-methyl benzimidazole to be attached at the C3, C7, and C12 positions of CA with varied alkyl chains from methyl to octyl (**1**–**8**) at the C24 position to establish a structure-activity relationship (SAR) (Figure 2). For synthesis, methyl to octyl-alkyl chains were incorporated at the C24 position of the CA via an ester linkage using the reported methods (Figure 2) [28]. Next, the respective cholic esters (**10a**–**10h**) were treated with chloroacetic anhydride in the presence of DMAP to yield chloroacetylated cholic esters (**11a**–**11h**). The *N-methyl* benzimidazole moiety was then tethered via a nucleophilic substitution reaction on chloroacetylated cholic esters to obtain the final amphiphiles (**1**–**8**). The intermediates and final molecules were characterized with ^1^H NMR spectroscopy, and the purity of the final molecules was analyzed via reverse phase HPLC (see Supplementary Materials).

### 2.2. Antibacterial Activities of CA-Benzimidazole Amphiphiles

We screened all the amphiphiles against different Gram-positive and Gram-negative bacteria using a broth dilution assay and calculated the minimum inhibitory concentrations at which 99% bacterial death was observed. Bacteria (~10^6^ CFU/mL) in a 96-well plate were treated with amphiphiles using the serial dilution method from 256 to 1 μg/mL. After incubation of the cultures for 16 h at 30 °C, we measured the OD of the solution at 600 nm to determine the MIC_99_. Structure activity investigations revealed that amphiphiles are more active against Gram-positive bacteria than Gram-negative bacteria (Table 1). SAR studies demonstrated that amphiphiles bearing butyl to octyl chains showed antibacterial activity against *S. aureus*. Interestingly, at MIC_99_ of the amphiphiles having hexyl and heptyl chains accounted for 16 μg/mL against *S. aureus* and 2 μg/mL against *S. oralis*, whereas amphiphiles with short-chain molecules were found to be inactive against different pathogens (Table 1). Therefore, for further studies, a CABI-**6** amphiphile with a hexyl chain was employed.

### 2.3. CABI-6 Amphiphile Is Bactericidal

**Figure 3:**
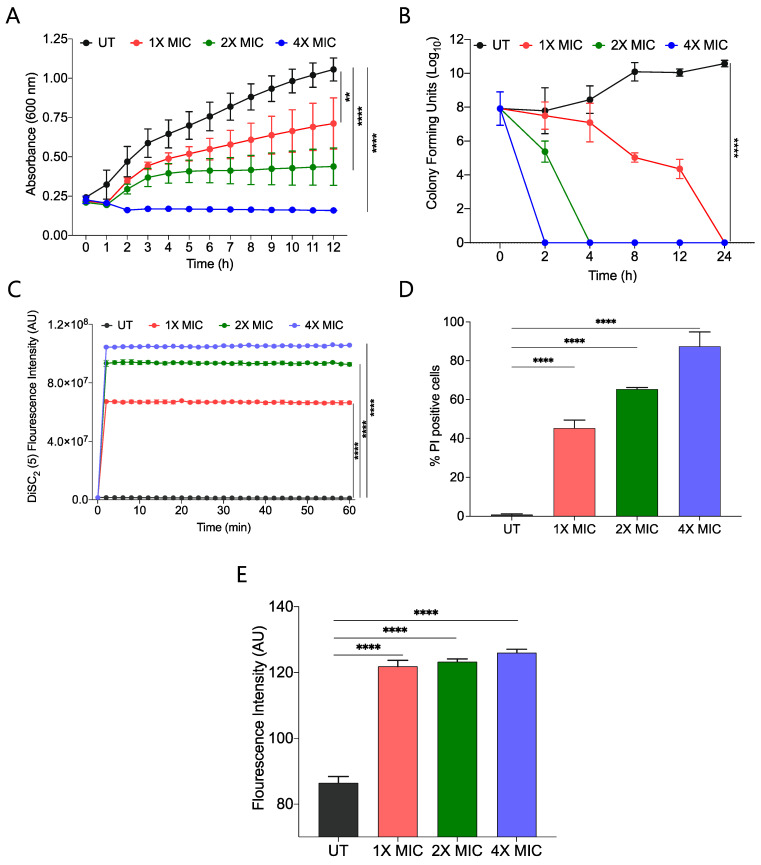
(**A**) Growth kinetic studies of *S. aureus* in presence of CABI-**6** confirm the dose-dependent antibacterial potency of CABI-**6**. (**B**) Time-kill kinetics showing dose-dependent bactericidal activity of CABI-**6** against *S. aureus*. (**C**) Fluorescence-based assay displaying the membrane permeabilizing property of CABI-**6** against *S. aureus*. (**D**) Dose-dependent percentage increase in the number of propidium iodide (PI)-positive bacterial cells endorse the membrane disruptive property of CABI-**6**. (**E**) Fluorescence-based assay confirms bactericidal activity by increasing the production of reactive oxygen species. Data were analysed by ANOVA (**** *p* < 0.0001; ** *p* < 0.001).

We performed a growth kinetic study of *S. aureus* using three different concentrations, 1X, 2X, and 4X MIC_99_ of active amphiphile (CABI-**6**) and observed a dose-dependent activity of CABI-**6** against *S. aureus*. Treatment at 1X and 2X MIC_99_ of CABI-**6** restrict the bacterial growth, and 4X MIC_99_ of CABI-**6** did not allow for the growth of the bacteria (Figure 3A). To investigate the bacteriostatic or bactericidal nature of the CABI-**6** amphiphile, we conducted a time-dependent kill kinetic study against *S. aureus* and quantified the colony forming units (CFUs) of CABI-**6** treated *S. aureus* at different time points. We witnessed dose-dependent bactericidal activity of CABI-**6** as 1X MIC_99_ of CABI-**6** could clear the bacteria within 24 h, and 2X MIC_99_ of CABI-**6** took 4 h to kill the bacteria completely (Figure 3B). In contrast, 4X MIC_99_ showed absolute bacterial inhibition after 2 h treatment.

### 2.4. CABI-6 Amphiphile Can Disrupt the Bacterial Membranes

To decipher the membrane-disruptive property of CABI-**6**, we conducted a fluorescence-based assay using 3,3-diethylthiadicarbocyanine iodide dye [DiSC_2_(5)] that can accumulate and get quenched in bacterial membranes [35]. We stained *S. aureus* cells with DiSC_2_(5) dye and treated them with different concentrations of CABI-**6**. Among the different treatment groups, 4X MIC_99_ showed a sharp increase in fluorescence, suggesting that CABI-**6** can disrupt the bacterial cell membrane potential (Figure 3C). To validate the membrane-disruptive property of CABI-**6**, we performed a propidium iodide (PI) uptake assay with where PI can stain nucleic acids of membrane raptured cells as it is a membrane impermeable dye [36]. We stained the bacterial cells after their treatment with different concentrations of CABI-**6** and measured the percentage of PI-positive cells using flow cytometry. We witnessed ~50% PI-positive bacterial cells at a 1X MIC_99_ treatment of CABI-**6**, whereas a treatment with 2X and 4X MIC_99_ showed ~65 and ~90% PI-positive bacterial cells, respectively (Figure 3D).

Intracellularly released reactive oxygen species (ROS) play a key role in membrane lipid peroxidation, protein damaging, and importantly mitochondrial dysfunction [37]. To study the impact of CABI-**6** on ROS production, we performed a fluorescence-based assay using 2′,7′-Dichlorofluorescein diacetate (DCFH-DA). *S. aureus* cells were treated with 1X, 2X, and 4X MIC_99_ of CABI-**6** for 1 h, and released ROS were quantified by DCF fluorescence intensity. We observed a multi-fold increase in fluorescence indicating an increase in the production of ROS upon treatment with CABI-**6** amphiphile (Figure 3E). Therefore, these results demonstrate that CABI-**6** can disrupt the bacterial membranes effectively and generate ROS that are responsible for bacterial death.

### 2.5. CABI-6 Can Eradicate the Preformed S. aureus Biofilms

**Figure 4:**
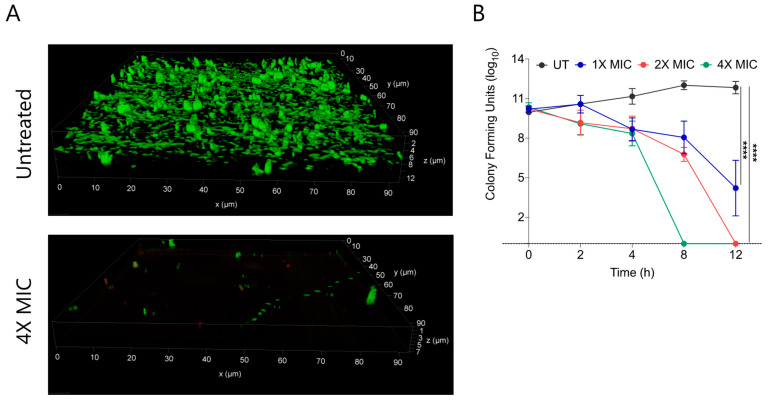
(**A**) Fluorescence micrographs of SYTO9/PI stained untreated and 4X MIC_99_ treated (CABI-**6**) *S. aureus* biofilms. (**B**) Ability of CABI-**6** potential to clear the *S. aureus* biofilms is confirmed by dose-dependent and time-dependent changes in CFUs. Data was analysed by ANOVA (**** *p* < 0.0001).

Biofilms are composed of extracellular polymeric substances (EPS) that provide a protective sheath to bacterial cells against external substances including antimicrobial agents. Bacterial biofilms are multi-layered entities of surface-associated cells that defend themselves from antibacterial agents. A prefabricated bacterial biofilm on medical devices and implants is linked to more than 60% of nosocomial infections. To test the biofilm eradication character of CABI-**6**, we placed an developed *S. aureus* bacterial biofilm on cover slips, and treated them with 1X, 2X, and 4X MIC_99_ of CABI-**6**. We used SYTO9 and PI to stain the untreated and treated biofilms, and fluorescence micrographs revealed that CABI-**6** can eradicate the *S. aureus* biofilm (Figure 4A). We further validated this data using CFU analysis and observed that 4X MIC_99_ treatment for 8 h was sufficient to clear the biofilms, whereas 2X MIC_99_ treatment can clear the biofilms within 12 h (Figure 4B).

### 2.6. CABI-6 can Mitigate the S. aureus-Mediated Murine Wound Infection 

**Figure 5:**
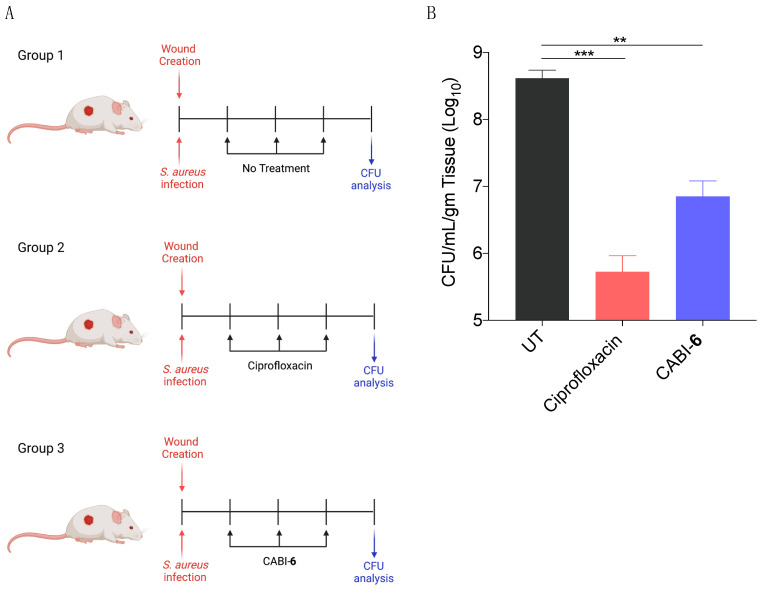
(**A**) Schema representing the experimental plan and different treatment groups (5 mice per group). (**B**) CFU analysis of *S. aureus* infected wounds post 3 days of treatment display that CABI-**6** can reduce the bacterial burden by >1.5-log as compared to untreated wounds. Data was analysed by ANOVA (*** *p* < 0.0001; ** *p* < 0.001).

Since *S. aureus* is associated with skin infections, we examined the potential efficacy of CABI-**6** against a murine wound infection model. On BALB/c mice, we made 1 cm^2^ circular wounds and infected them with *S. aureus* cells. We divided the mice into three groups (five mice per group) after 18 h of infection, and group 1 was assigned as untreated (Figure 5A). Group 2 mice were given ciprofloxacin (40 mg/kg), while group 3 animals were given CABI-**6** (40 mg/kg) at the infection site. Treatments were given three times a day for three days. After the completion of the experiment, we removed the skin tissue, homogenized it, plated it on agar plates, and counted the colony forming units. CABI-**6** therapy resulted in >1.5 log fold decrease in CFUs compared to the untreated control group (Figure 5B). These findings suggest that CABI-**6** is effective against *S. aureus*-mediated wound infection.

## 3. Experimental Section

### 3.1. Materials

Laboratory grade solvents used for chromatography were purchased from Rankem and were used without distillation. Cholic acid, dicyclohexylcarbodiimide (DCC), and anhydrous solvents used in synthesis including methanol, dichloromethane, ethyl acetate were obtained from Sigma-Aldrich, St. Louis, MO, USA. Materials including *p*-dimethyl amino pyridine (DMAP) were purchased from SpectroChem India, and *N*-methyl benzimidazole was obtained from Chemsworth. Thin layer chromatography (TLC) was carried out on aluminum coated with silica gel 60 GF 254 (Merck, Darmstadt, Germany), and stained with a methanolic solution of phosphomolybdic acid. Solvents used for HPLC were purchased from Thermo Fischer Scientific, Waltham, MA, USA. ^1^H-NMR spectra were recorded using a Bruker Avance 400 MHz spectrometer in CDCl_3_ and DMSO-d6. Chemical shifts (δ) are reported in ppm, and tetra methyl silane (TMS) was used as the internal standard. The splitting patterns were assigned as s, singlet; bs, broad singlet; d, doublet; triplet, t; and m, multiplet. The purity of the final molecules was checked with a reverse phase Waters HPLC system, equipped with an Octyl-80Ts C8 column (S0005) 4.6 × 250 mm (5 μm) with UV detection at 253 nm. An HPLC isocratic 10-min run program was set using mixtures of solvent A (0.1% trifluoracetic acid in water): solvent-B (0.1% trifluoracetic acid in acetonitrile) (2:8) at 25 °C with a flow rate of 0.5 mL/min.

### 3.2. Synthesis of Cholic Acid Esters (***10a***–***10h***)

Cholic acid esters were synthesized as per earlier reported reaction conditions [28].

### 3.3. Synthesis of Chloroacetylated Cholic Acid Esters (***11a***–***11h***)

Cholic acid ester (1 equivalent) was dissolved in anhydrous DCM and cooled over an ice bath. After cooling the mixture, DMAP (6 equivalent) was added to it and then it was allowed to stir for 15 min. Chloroacetic anhydride (6 equivalent.) was dissolved in DCM, and slowly added to the mixture. The reaction was shifted to room temperature after 30 min and allowed to stir. The progress of the reaction was monitored by TLC. After the completion of the reaction, it was diluted with DCM and washed with a saturated solution of sodium bicarbonate (200 mL × 2) and brine (200 mL × 2). The organic layer was collected separately and dried over sodium sulphate. Next, the organic layer was concentrated using a rotary evaporator to obtain the crude product. These intermediates were purified by column chromatography using silica (60–120 mesh size) as a stationary and petroleum ether/ethyl acetate as a mobile phase. The intermediates were characterized by ^1^H-NMR.

Compound **11a**. Yield: 97.2%, ^1^H NMR: (400 MHz, CDCl_3_) *δ*: 0.75 (s, 3 H), 0.82–2.33 (m, 37 H), 3.66 (s, 3 H), 4.02–4.11 (m, 6 H), 4.66 (m, 1 H), 5.03 (s, 1 H), 5.29 (s, 1 H).

Compound **11b**. Yield: 90.6%, ^1^H NMR: (400 MHz, CDCl_3_) *δ*: 0.78 (s, 3 H), 0.85–2.33 (m, 44 H), 4.04–4.16 (m, 8 H), 4.69 (m, 1 H), 5.06 (m, 1 H), 5.22 (s, 1 H).

Compound **11c**. Yield: 86.1%, ^1^H NMR: (400 MHz, CDCl_3_) *δ*: 0.77 (s, 3 H) 0.84–2.35 (m, 41 H), 4.02–4.13 (m, 8 H), 4.68 (m, 1 H), 5.06 (s, 1 H), 5.22 (s, 1 H).

Compound **11d**. Yield: 84.7%, ^1^H NMR: (400 MHz, CDCl_3_) *δ*: 0.90 (s, 3 H), 1.08–2.30 (m, 41 H), 4.03–4.10 (m, 8 H), 4.65 (m, 1 H), 5.03 (m, 1 H), 5.19 (s, 1 H).

Compound **11e**. Yield: 83.3%, ^1^H NMR: (400 MHz, CDCl_3_) *δ*: 0.75 (s, 3 H), 0.82–2.32 (m, 46 H), 4.04–4.12 (m, 8 H), 4.66 (m, 1 H), 5.03 (s, 1 H), 5.20 (s, 1 H).

Compound **11f**. Yield: 90.6%, ^1^H NMR: (400 MHz, CDCl_3_) *δ*: 0.82 (s, 3 H) 0.83–2.32 (m, 50 H), 4.04–4.11 (m, 8 H), 4.65 (m, 1 H), 5.03 (s, 1 H), 5.19 (s, 1 H).

Compound **11g**. Yield: 95.6%, ^1^H NMR: (400 MHz, CDCl_3_) *δ*: 0.77 (s, 3 H), 0.84–2.35 (m, 51 H), 4.05–4.15 (m, 8 H), 4.63–4.70 (m, 1 H), 5.06 (s, 1 H), 5.22 (s, 1 H). 

Compound **11h**. Yield: 78.1%, ^1^H NMR: (400 MHz, CDCl_3_) *δ*: 0.76 (s, 3 H) 0.83–2.09 (m, 52 H), 4.03–4.13 (m, 8 H), 4.68 (m, 1 H), 5.04 (s, 1 H), 5.21 (s, 1 H).

### 3.4. Synthesis of CABI Amphiphiles

Choloacetylated cholic acid ester (1 eq.) was dissolved in ethyl acetate and *N*-methyl benzimidazole (6 eq.) was added to the mixtures. The reaction mixtures were refluxed at 70 °C for 72 h. After the completion of the reaction, mixtures were cooled, and precipitated using *n*-hexane and diethyl ether. To yield pure amphiphiles, repeated washes of *n*-hexane and diethyl ether were given. CABI amphiphiles were characterized via ^1^H-NMR spectroscopy, high resolution mass spectrometry (HRMS) and the purity of the amphiphiles was calculated by reverse phase HPLC.

CABI-**1**: ^1^H NMR (DMSO-d_6_, 400 MHz): 0.71 (s, 3 H), 0.76–2.49 (m, 34 H), 3.64 (s, 3 H), 4.11 (s, 3 H), 4.25 (s, 6 H), 4.89 (m, 1 H), 5.12 (m, 1 H), 5.83 (m, 1 H), 6.09–6.15 (m, 1 H), 6.24–6.30 (m, 5 H), 7.62–8.40 (m, 15 H). IR (cm^−1^): 3366, 2951, 2361, 1741, 1217, 770. HRMS: Calculated (M + H^+^): 1047.4312; Observed: 1047.4171, HPLC: 3.617 min, >99%.

CABI-**2**: ^1^H NMR (MeOD-d_4_, 400 MHz): 0.86 (s, 3 H), 0.88–2.49 (m, 35 H), 4.11 (t, 2 H, *J* = 6 Hz), 4.18 (s, 3 H), 4.29 (bs, 6 H), 5.15 (s, 1 H), 5.35 (m, 1 H), 5.6–5.7 (m, 1 H), 5.82–6.01 (m, 3 H), 6.01–6.1 (m, 1 H) 7.31–7.37 (m, 2 H), 7.57–8.18 (m, 13 H). IR (cm^−1^): 3378, 2948, 2361, 1737, 1572, 1465, 1365, 1223, 1033, 755. HRMS: Calculated (M + H^+^) 1061.4469; observed: 1061.4261. HPLC: 3.636 min, >98.3%.

CABI-**3**: ^1^H NMR (MeOD-d_4_, 400 MHz): 0.74–2.23 (m, 49 H), 3.99 (t, 2 H, *J* = 6 Hz), 4.14 (s, 3 H), 4.17 (s, 6 H), 4.78 (s, 1 H), 5.03 (s, 1 H), 5.23 (s, 1 H), 5.50–5.60 (m, 1 H), 5.70–5.87 (m, 4 H), 5.91–6.01 (m, 1 H), 7.25–7.40 (m, 1 H), 7.53–7.98 (m, 14 H). IR (cm^−1^): 3379, 2965, 2359, 1736, 1572, 1465, 1382, 1224, 1033, 755. HRMS: Calculated (M - Cl) 1039.4861; observed: 1039.4875. HPLC: 3.632 min, >99%.

CABI-**4**: ^1^H NMR (MeOD-d_4_, 400 MHz): 0.86–2.37 (m, 42 H), 4.12 (t, 2 H, *J* = 6 Hz), 4.18 (m, 3 H), 4.26 (s, 6 H), 5.15 (s, 1 H), 5.35 (s, 1 H), 5.60–5.70 (m, 1 H), 5.82–5.87 (m, 4 H), 6.01–6.11 (m, 1 H), 7.40–7.5 (m, 1 H), 7.65–8.10 (m, 14 H). IR (cm^−1^): 3379, 2968, 2361, 1739, 1572, 1463, 1354, 1215, 962, 755. HRMS: Calculated (M + H^+^): 1089.4782; Observed: 1089.4565. HPLC: 3.669 min, >98%.

CABI-**5**: ^1^H NMR (MeOD-d_4_, 400 MHz): 0.86 (s, 3 H), 0.88–2.40 (m, 39 H), 4.11 (t, 2 H, *J* = 6 Hz), 4.18 (s, 3 H), 4.29 (s, 6 H), 5.35 (s, 1 H), 5.64–5.71 (m, 1 H), 5.81–5.99 (m, 3 H), 7.31–7.40 (m, 2 H), 7.59–8.18 (m, 13 H). IR (cm^−1^): 3381, 2959, 2361, 1738, 1572, 1463, 1216, 961, 755. HRMS: Calculated (M + H^+^) 1103.4628, 1103.4744. HPLC: 3.964 min, >98%.

CABI-**6**: ^1^H NMR (MeOD-d_4_, 400 MHz): 0.87–2.41 (m, 48 H), 4.13 (t, 2 H, *J* = 6 Hz), 4.18 (s, 3 H), 4.29 (s, 6 H), 5.16 (s, 1 H), 5.35 (s, 1 H), 5.6–5.7 (m, 1 H), 5.82–5.86 (m, 4 H), 6.0–6.1 (m, 1 H), 7.30–7.40 (m, 1 H), 7.65–8.13 (m, 14 H). IR (cm^−1^): 3375, 2958, 2360, 1737, 1572, 1463, 1382, 1220, 962, 752. HRMS: Calculated (M + Na^+^) 1139.4922; observed: 1139.4569. HPLC: 3.89 min, >98%.

CABI-**7**: ^1^H NMR (MeOD-d_4_, 400 MHz): 0.85–2.42 (m, 57 H), 4.12 (t, 2 H, *J* = 6 Hz), 4.19 (s, 3 H), 4.30 (s, 6 H), 5.12 (s, 2 H), 5.32 (s, 1 H), 5.60–5.70 (m, 1 H), 5.84–5.89 (m, 5 H), 7.66–813 (m, 15 H). IR (cm^−1^): 3385, 2969, 2360, 1739, 1571, 1463, 1382, 1215, 962, 961, 755. HRMS: calculated (M + H^+^): 1131.5251; observed: 1131.5068. HPLC: 4.013 min, >98%.

CABI-**8**: ^1^H NMR (MeOD-d_4_, 400 MHz): 0.85–2.39 (m, 58 H), 4.12 (t, 2 H, *J* = 6 Hz), 4.19 (s, 3 H), 4.30 (s, 6 H), 5.15 (s, 2 H), 5.35 (s, 2 H), 5.79–5.81 (m, 1 H), 5.84–6.01 (m, 6 H), 7.66–813 (m, 15 H). IR (cm^−1^): 3381, 2979, 2360, 1738, 1572, 1463, 1382, 1217, 962, 755. HRMS: calculated (M - Cl): 1109.5644; observed: 1109.5660. HPLC: 4.049 min, >98%.

### 3.5. Material and Methods

LB Broth (M1245), Tryptone soya broth (TSB) (M011), and Bacteriological agar (GRM026) were used for culturing different strains, which were purchased from HiMedia, Maharashtra, India. Ciprofloxacin (17850), propidium iodide (P1470), 3,3-Diethylthiadicarbocyanine, iodide (17375), and 2′,7′-Dichlorofluorescein diacetate (35845) were procured from Sigma-Aldrich, St. Louis, MO, USA. LIVE/DEAD^®^ BacLightTM bacterial viability and counting kit from Invitrogen, Waltham, MA, USA, and *Streptococcus aureus* (MTCC737), *Streptococcus pneumoniae* (MTCC1936), *Bacillus subtilis* (MTCC441), *Escherichia coli* (MTCC443), and *Pseudomonas aeruginosa* (MTCC1688) were purchased from the MTCC (Chandigarh, India).

Minimum inhibitory concentration. To calculate the minimum inhibitory concentrations (MIC_99_) of the CABI series, we used a microdilution method which was approved by the CLSI (Clinical and Laboratory Standards Institute). We then serially diluted synthesized compounds in a 96 well plate from a concentration of 128 μg/mL to 0.25 μg/mL and added 100 μL of *S. aureus* cells (5^10^5^ cells/mL) in each well. These plates were incubated for 16–18 h at 37 °C. After incubation optical density was observed at 600 nm by Spectramax M5 multimode microplate reader (Molecular Devices, Sunnyvale, CA, USA) to find out the MIC_99_ of each compound.

Growth curve kinetics. A specific growth kinetic is a characteristic feature of individual bacteria. In this case, log phase *S. aureus* cells diluted up to 10^6^ CFU/mL in LB media and 100 μL of working suspension was added to a 96 well plate. The different concentrations of CABI-**6** (1X, 2X and 4X MIC_99_) were poured in the 96 well plate (in 100 μL). The growth kinetics of all samples were observed by using Spectramax M5 multimode microplate reader (Molecular Devices, Sunnyvale, CA, USA) at 600 nm.

Time-kill kinetics. A sole colony of *S. aureus* was inoculated in LB media for 6–8 h at 37 °C to prepare a culture of ~0.5 OD. These mid log phase cells further diluted to 0.1 OD (~10^7^ cells/mL) in 1X PBS (pH-7.4) and 100 μL of these cells were treated with different concentration of CABI-**6** (at 1X, 2X, and 4X MIC_99_) with untreated cells. These cells further incubated for different time points (0 h, 2 h, 4 h, 8 h, 12 h, and 24 h) at 37 °C to illustrate the bactericidal activity of CABI-**6**. At different time points, treated and untreated cells were serially diluted and plated on the LB agar plate. Finally, the bacterial colonies were counted, and results were represented in log_10_CFUs/mL vs. time.

DiSC_2_(5) fluorescence assay. To quantify the role of CABI-**6** in depolarizing the membrane potential of bacterial cells, we used a dye-based assay. *S. aureus* cells were cultured until log phase and harvested at 5000 rpm for 5 min. These cells were resuspended into 1X PBS to adjust the OD 0.1 (working concentration 10^7^ CFU/mL). Next, suspension culture was stained with DiSC_2_(5) (a final concentration equals to 100 μM) dye and fluorescence intensity was observed after every 15 min until the saturation of fluorescence. After obtaining saturation by the dye, we treated these cells with KCl (100 mM) to stabilize the stained cells. Furthermore, stained cells were incubated with different concentrations of CABI-**6** for 15 min at 37 °C. After incubation, we set kinetics for the 60 min at excitation 637 nm and emission 670 nm at the 2 min interval.

ROS assay. Release of ROS is the characteristic feature which suggested the damaging of bacterial membrane. To decipher the role CABI-**6** in ROS release, we cultured *S. aureus* cells until log phase and treat these cells with the different concentrations of CABI-**6** (1X, 2X and 4X MIC_99_) and followed by washing in PBS. Next, these treated cells stained with the DCFH-DA dye for 1 h. Untreated and treated stained cells were washed with PBS to remove the traces of DCFH-DA dye and solvent of each sample subjected to measured DCF fluorescence intensity at excitation 485 nm and emission 535 nm using Spectramax M5 multimode microplate reader (Molecular Devices, Sunnyvale, CA, USA).

Membrane permeabilization assay. The secondary culture of *S. aureus* was harvested at 5000 rpm for 5 min, and cells resuspended into 1X PBS. The working concentration of PBS resuspended cells were adjusted to 0.1 OD (10^7^ cells/mL). After that, cells were treated with a different concentration of CABI-**6** (1X, 2X and 4X MIC_99_) for 1 h and subjected to washing. Both untreated and treated washed cells were stained with propidium iodide (a final concentration10 μM/mL) for 10 min, and after washing, cells were resuspended into 100 μL. The percentage of PI positive cells was calculated by flowcytometry (BD FACS Verse flow cytometer (BD Biosciences, San Jose, CA, USA) in PI channel.

Biofilm degradation assay. For the biofilm degradation/disruption study, we prepared biofilms on cover-glass (18 mm × 18 mm). First, we added sterile coverslips in 6 well plates with 3 mL of TSB media (with 1% glucose). The mid log phase *S. aureus* cells were mixed, and the final OD adjusted to 0.1 in each well and then plates were incubated at 37 °C. The media were replenished after every 48 h, and the biofilms grown for 6 days. On day 6, when biofilms were completely grown, we treated these preformed biofilms with the different concentrations of CABI-**6** for different time points (0 h, 2 h, 4 h, 8 h, and 12 h) and quantified the data in log_10_CFUs/mL vs. time. For confocal imaging, PBS washed untreated and treated biofilms were stained with SYTO9/PI dye for 15 min and fixed with 4% PFA (paraformaldehyde). The imaging was conducted using the 63X oil objective of the inverted confocal microscope (Leica TCS SP8, Leica Germany, Munich, Germany), and by using fluorescein isothiocyanate (FITC for SYTO9, green) channel and tetramethylrhodamine isocyanate (TRITC for PI, red) channel.

In-vivo study (wound infection model). BALB/c male mice of 6–8 weeks (average weight 20 gm) were used to test the efficacy of CABI-**6** against wound infection. The hair from each group’s mice was trimmed from the dorsal right plank and the skin area was cleaned with veet cream. The cleaned surface was further sterilized with the povidone iodine followed by 70% alcohol. A wound of about 1 cm was created with scissors and forceps and infected with *S. aureus* (10^6^ cells/mice in 20 μL). After 18 h of infection, saline was applied to Group 1 (control) mice, and mice Groups 2 and 3 were treated with 40 mg/kg dose of ciprofloxacin and CABI-**6**, respectively, three time a day with treatment was continuing for 3 days. On day 4, mice were sacrificed, skin tissue samples collected in PBS for homogenization, plated on the agar plate, and then incubated for 16–18 h at 37 °C. The bacterial burden was calculated by counting the CFUs.

## 4. Conclusions

In summary, we have designed and synthesized a series of eight new cholic acid-derived amphiphiles where hydroxyl groups were tethered with *N*-methyl benzimidazole, and varying alkyl chains were conjugated at the C24 carboxylic acid of the cholic acid. Antibacterial screening results of these amphiphiles suggested that increasing the alkyl chain at the C24 position can improve antibacterial properties against *S. aureus*. Among the series, we have selected CABI-**6** (hexyl substituted amphiphile) as the potent antibacterial molecule. Mechanistically, CABI-**6** can hamper the bacterial cell membrane and cause bacterial death in a dose-dependent manner. In addition, CABI-**6** increases the production of ROS, which is the key mediator of bacterial death. Moreover, CABI-**6** can clear the preformed bacterial biofilm on cover slips. In addition, CABI-**6** can eradicate *S. aureus*-mediated wound infections. In the future, fine chemical tuning of CABI-**6** can be performed to create the next-generation of antibacterial agents.

## Figures and Tables

**Table 1:** Antibacterial properties of synthesized amphiphiles against different Gram-positive and Gram-negative bacteria.

Minimum Inhibitory Concentration (MIC_99_) (μg/mL)								
	Gram-Positive Strains				Gram-Negative Strains			
	*S. aureus*	*S. pneumoniae*	*B. subtilis*	*S. oralis*	*E. Coli*	*P. aeruginosa*	*K. pneumoniae*	*S. typhimurium*
CABI-**1**	>128	>128	>128	8	>128	>128	>128	>128
CABI-**2**	128	128	128	4	>128	>128	>128	>128
CABI-**3**	128	128	128	4	>128	128	>128	>128
CABI-**4**	32	32	64	2	>128	64	>128	>128
CABI-**5**	32	32	128	2	>128	32	>128	>128
CABI-**6**	16	32	128	2	>128	32	>128	>128
CABI-**7**	16	32	64	2	>128	16	>128	128
CABI-**8**	32	32	32	2	>128	32	>128	>128

## Data Availability

The characterization spectra including ^1^H NMR, HRMS, IR and HPLC chromatographs contained within supplementary information.

## Supplementary Materials

The following supporting information can be downloaded at: https://www.mdpi.com/article/10.3390/molecules27113501/s1. The characterization spectra including ^1^H NMR, HRMS, IR and HPLC chromatographs contained within supplementary information.
