# Supplementary material for: N-methyl Benzimidazole Tethered Cholic Acid Amphiphiles Can Eradicate S. aureus-Mediated Biofilms and Wound Infections
Source: Molecules. 2022 May 30;27(11):3501. doi: 10.3390/molecules27113501 (PMC9182351; doi:10.3390/molecules27113501)
Supplement: Supplementary file 1 [file molecules-27-03501-s001.zip › molecules-1686548-supplementary.pdf]

Supporting Information

N-methyl Benzimidazole Tethered Cholic Acid Amphiphiles Can Eradicate *S. aureus*-Mediated Biofilms and Wound Infections

Himanshu Kakkar [1], Nalini Chaudhary [2], Devashish Mehta [2], Varsha Saini [2], Shallu Maheshwari [1], Jitender Singh [1], PreetiWalia [2,*] and Avinash Bajaj [2,*]

1 Lord Shiva College of Pharmacy, Near Civil Hospital, Sirsa 125055, Haryana, India; himanshukakkar1670@gmail.com (H.K.); shallumaheshwari180@gmail.com (S.M.); saggujittu@gmail.com (J.S.)
2 Laboratory of Nanotechnology and Chemical Biology, Regional Centre for Biotechnology, NCR Biotech Science Cluster, 3rd Milestone, Faridabad-Gurgaon Expressway, Faridabad 121001, Haryana, India; nalinichaudhary2311@gmail.com (N.C.); devashish.mehta@rcb.res.in (D.M.); varsha.phd19@rcb.res.in (V.S.)
* Correspondence: preetiwalia_chem@yahoo.com (P.W.); bajaj@rcb.res.in (A.B.)

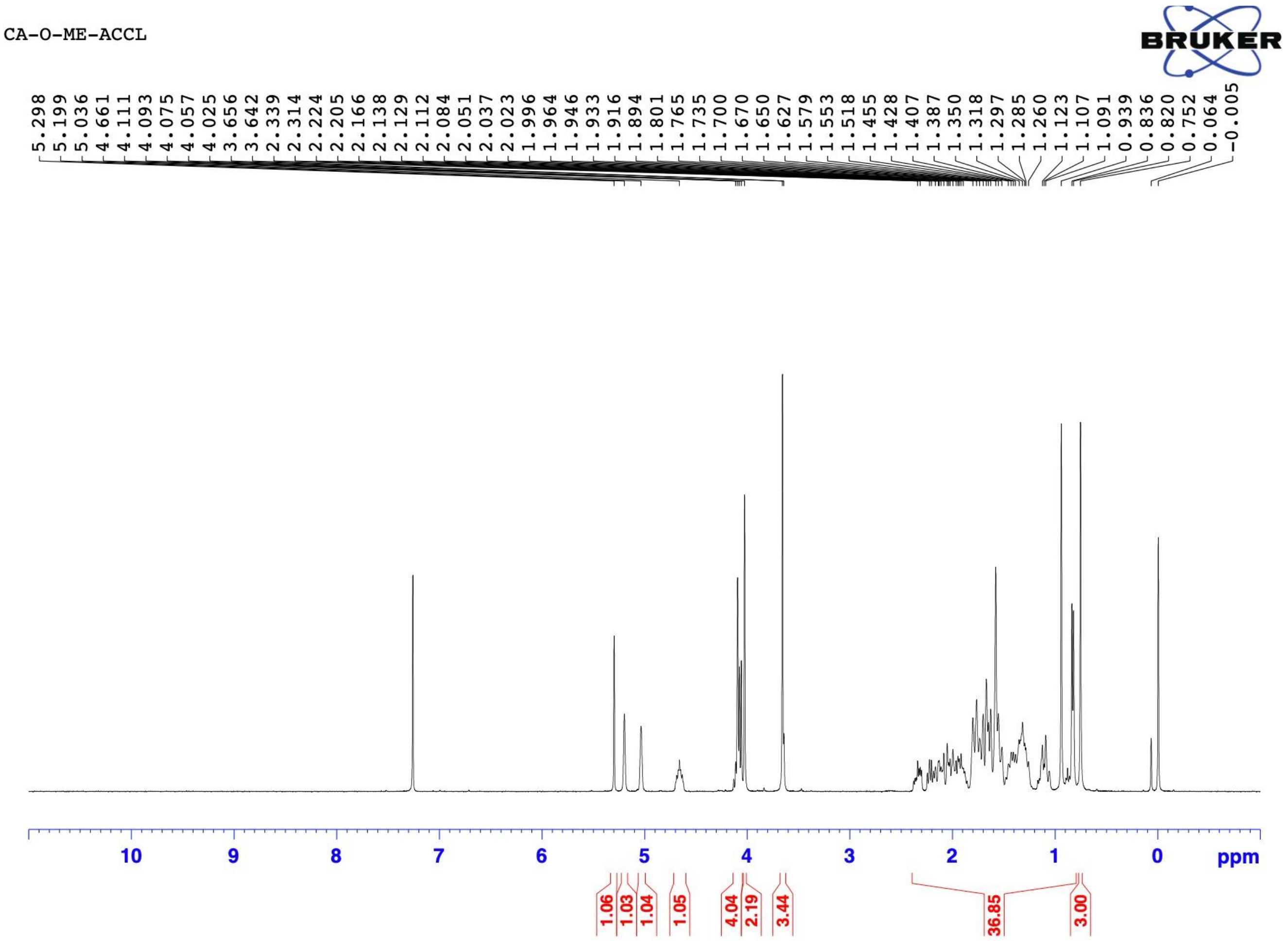

Figure S1. ^{1}H NMR spectrum of **11a**.

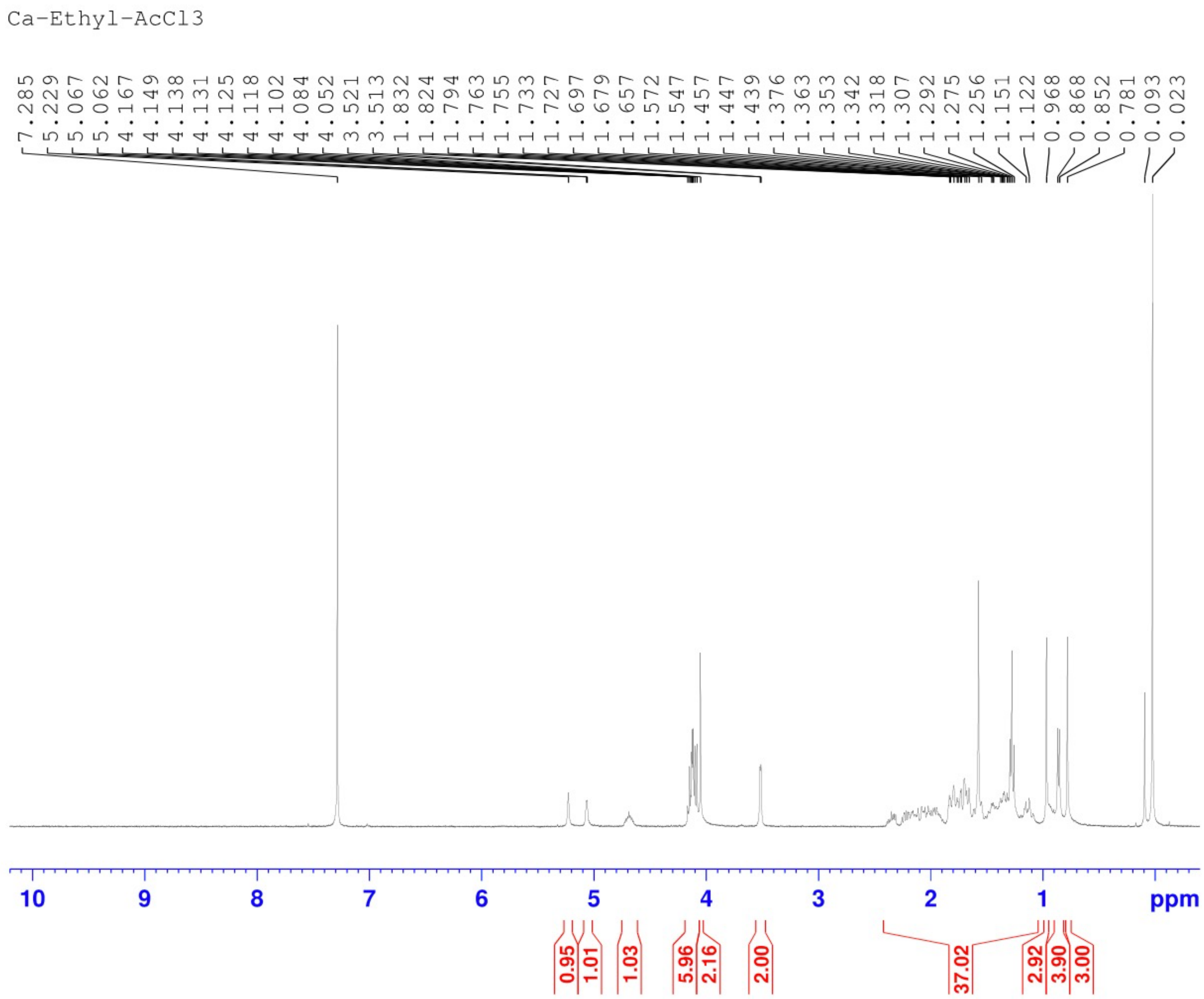

Figure S2. ^{1}H NMR spectrum of **11b**.

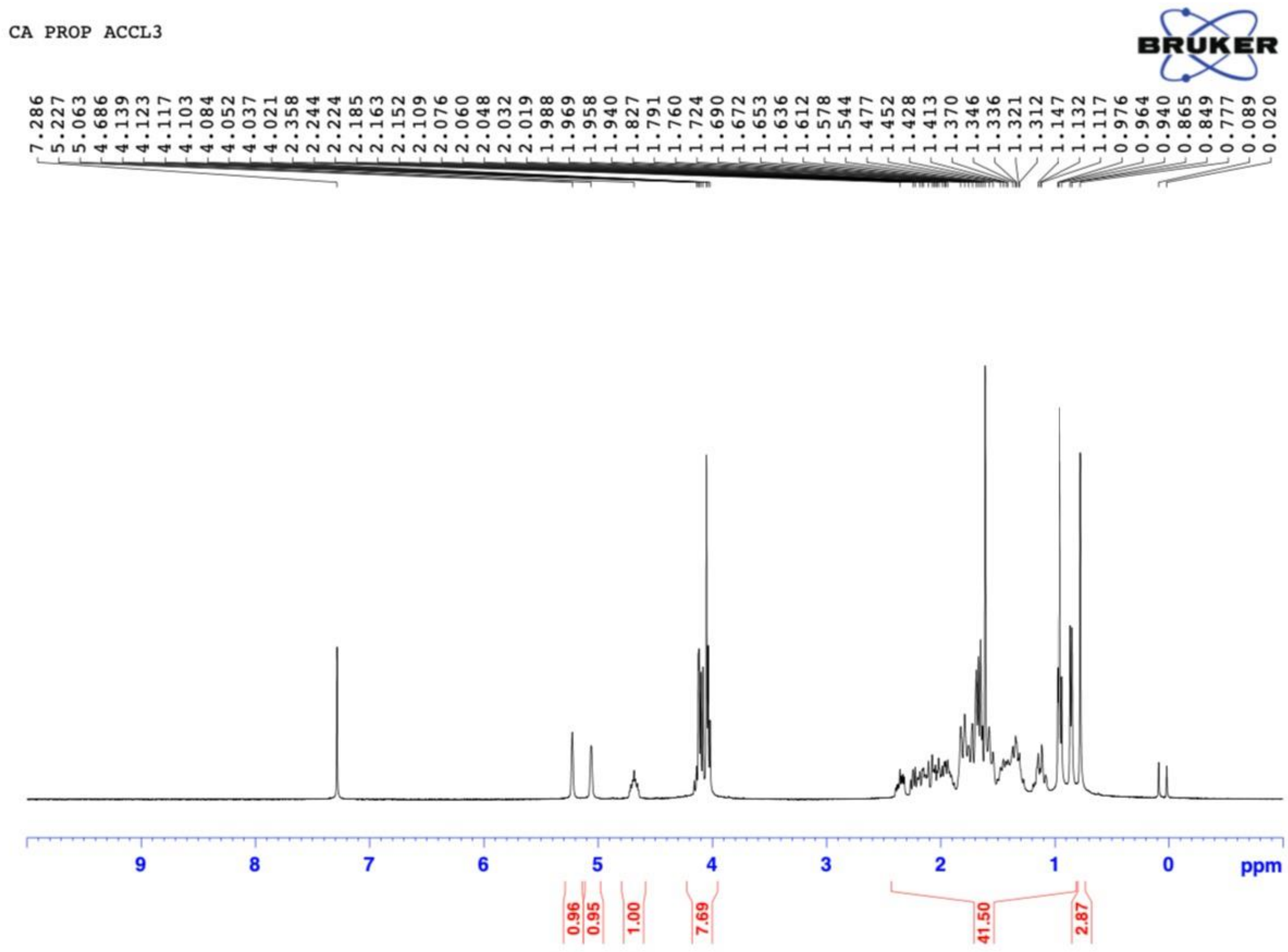

Figure S3. ^{1}H NMR spectrum of **11c**.

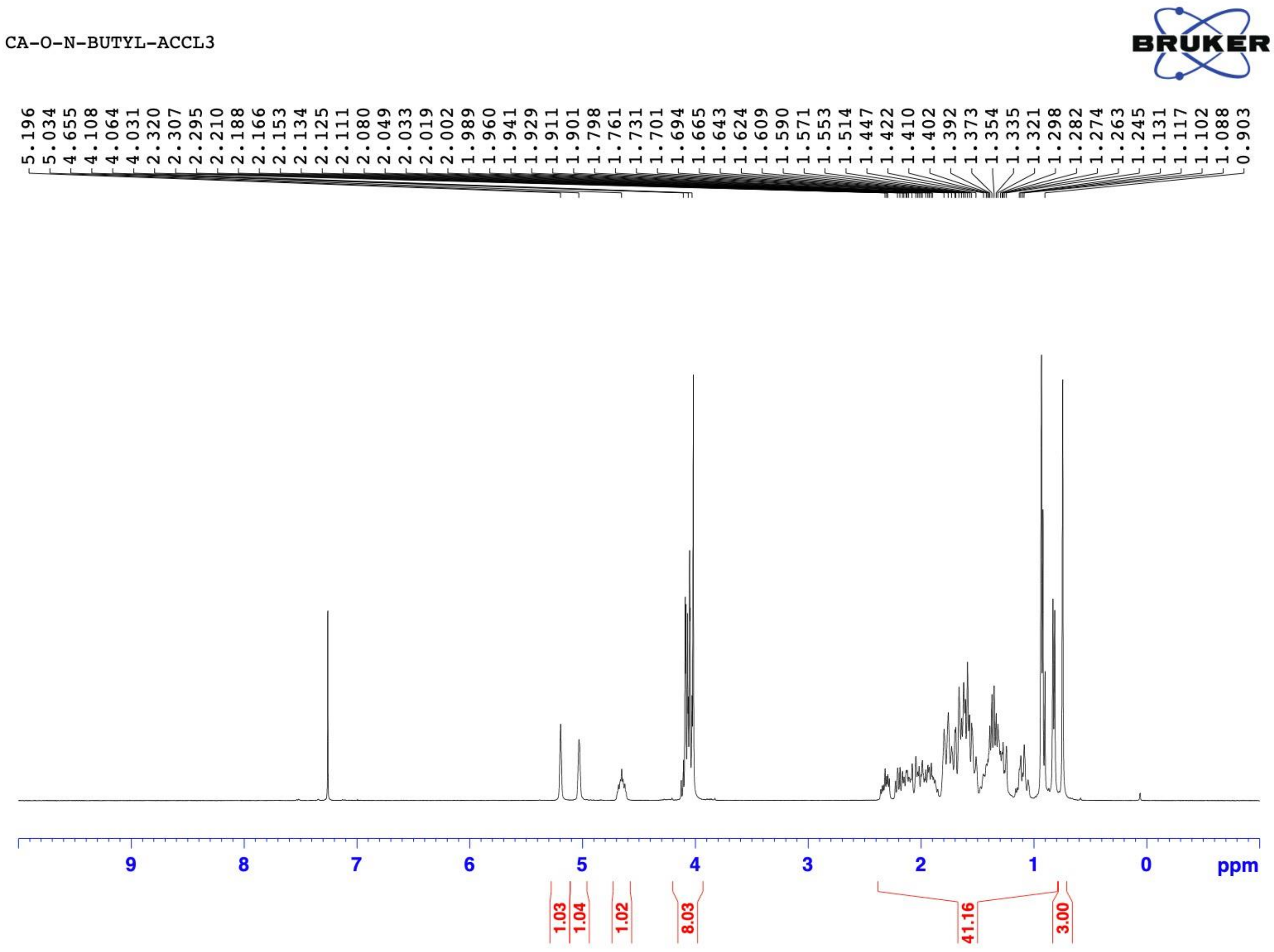

Figure S4. ^{1}H NMR spectrum of **11d**.

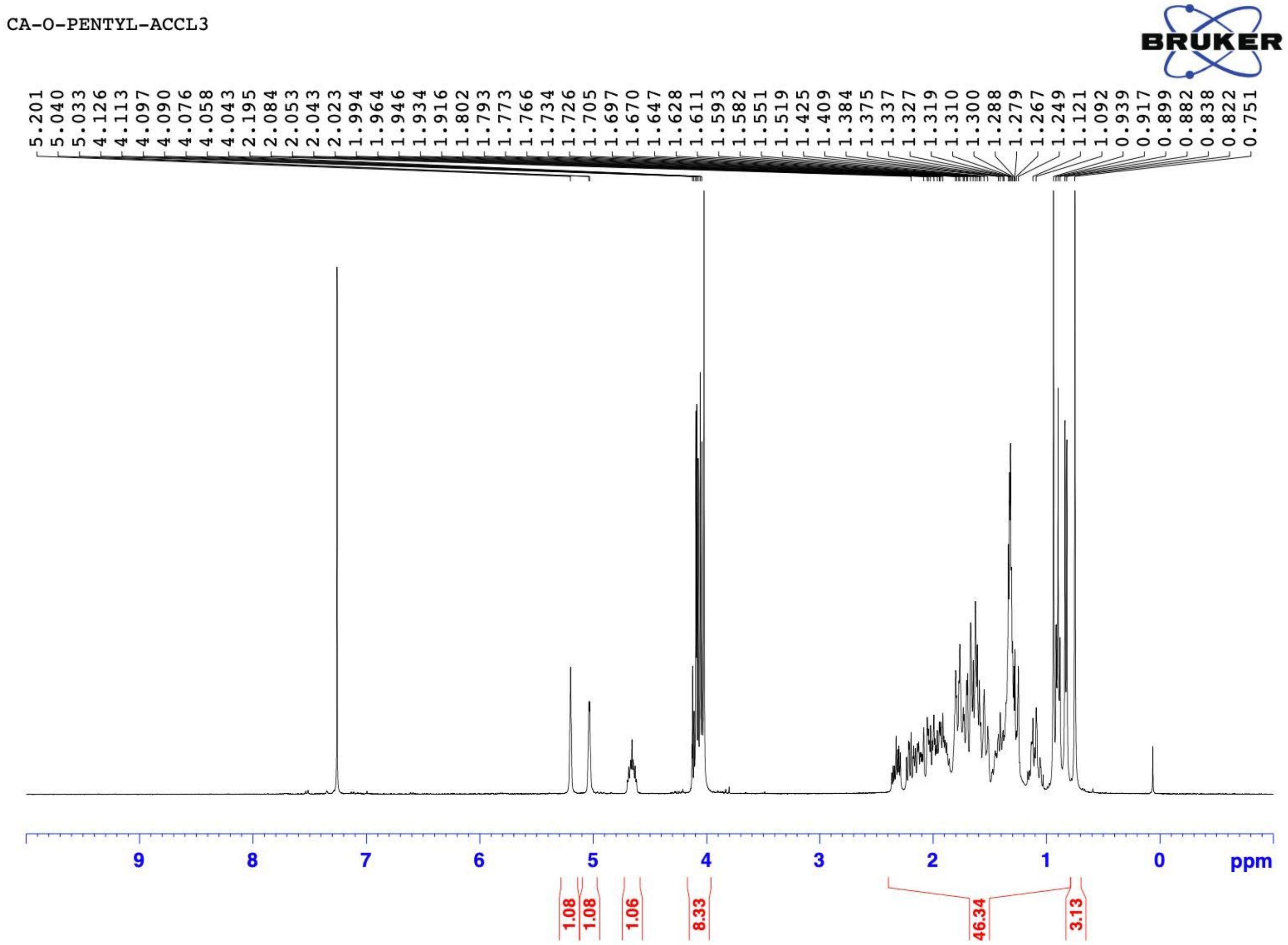

Figure S5. ^{1}H NMR spectrum of **11e**.

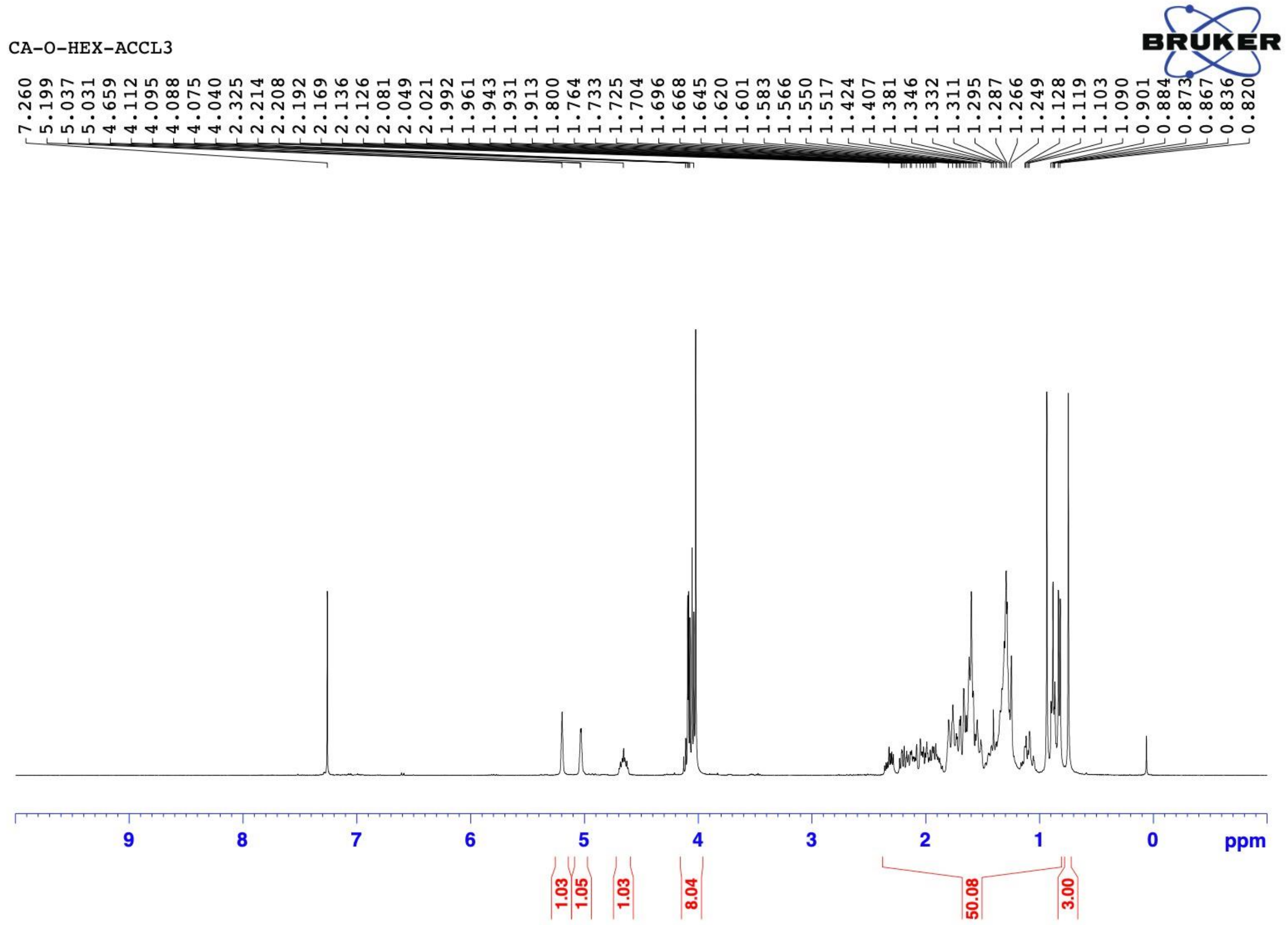

Figure S6. ^{1}H NMR spectrum of **11f**.

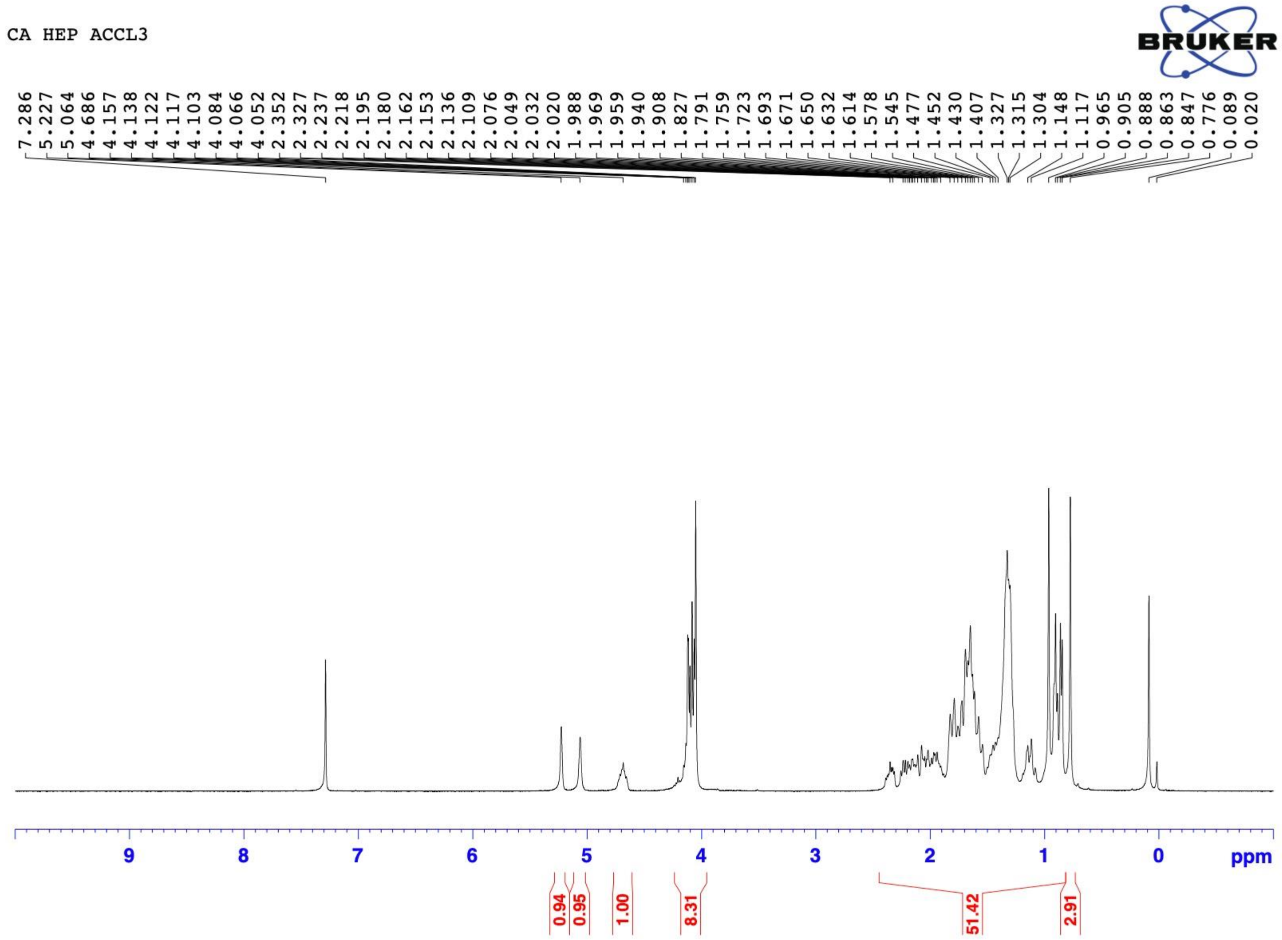

Figure S7. ^{1}H NMR spectrum of **11g**.

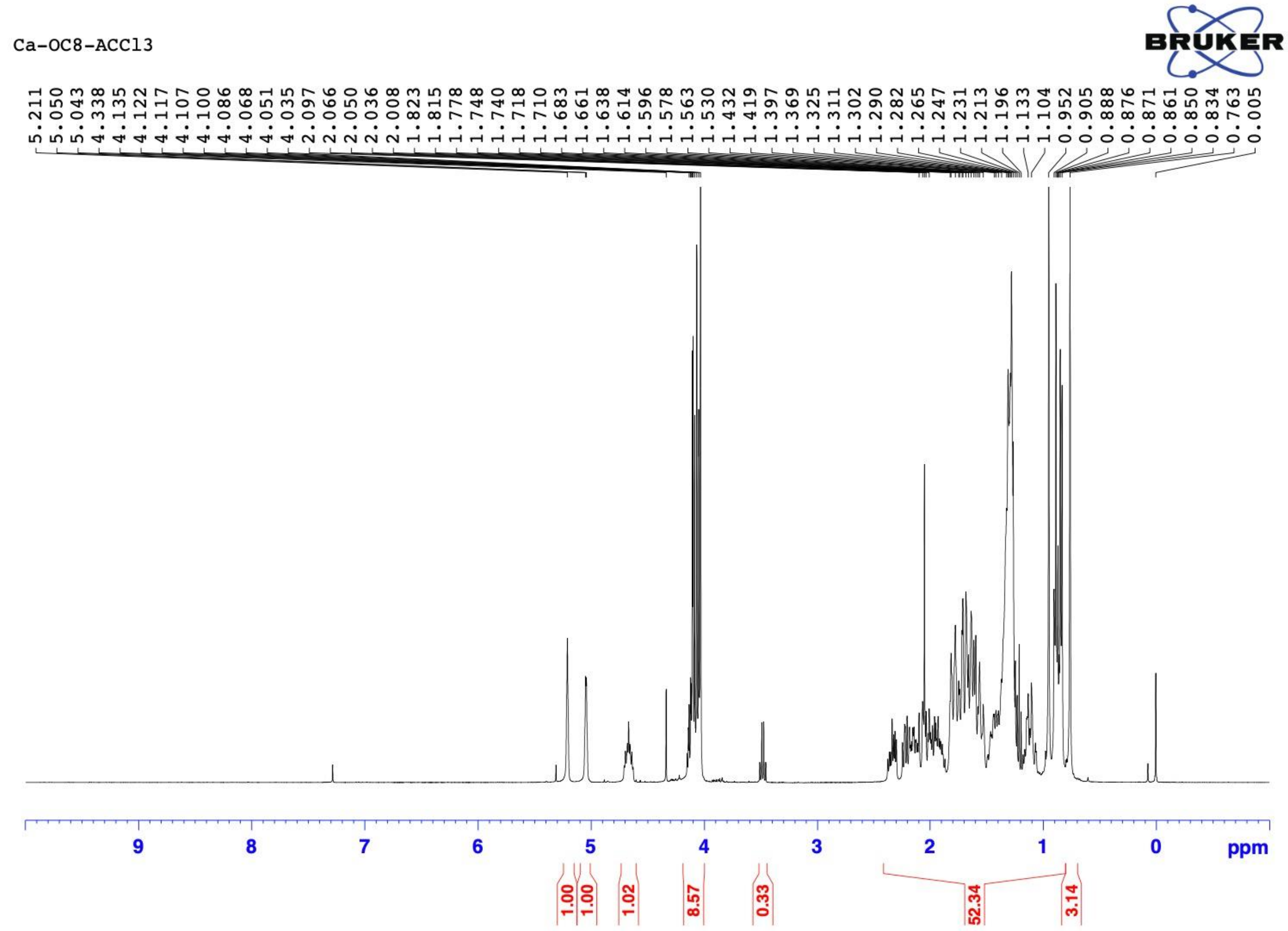

Figure S8. ^{1}H NMR spectrum of **11h**.

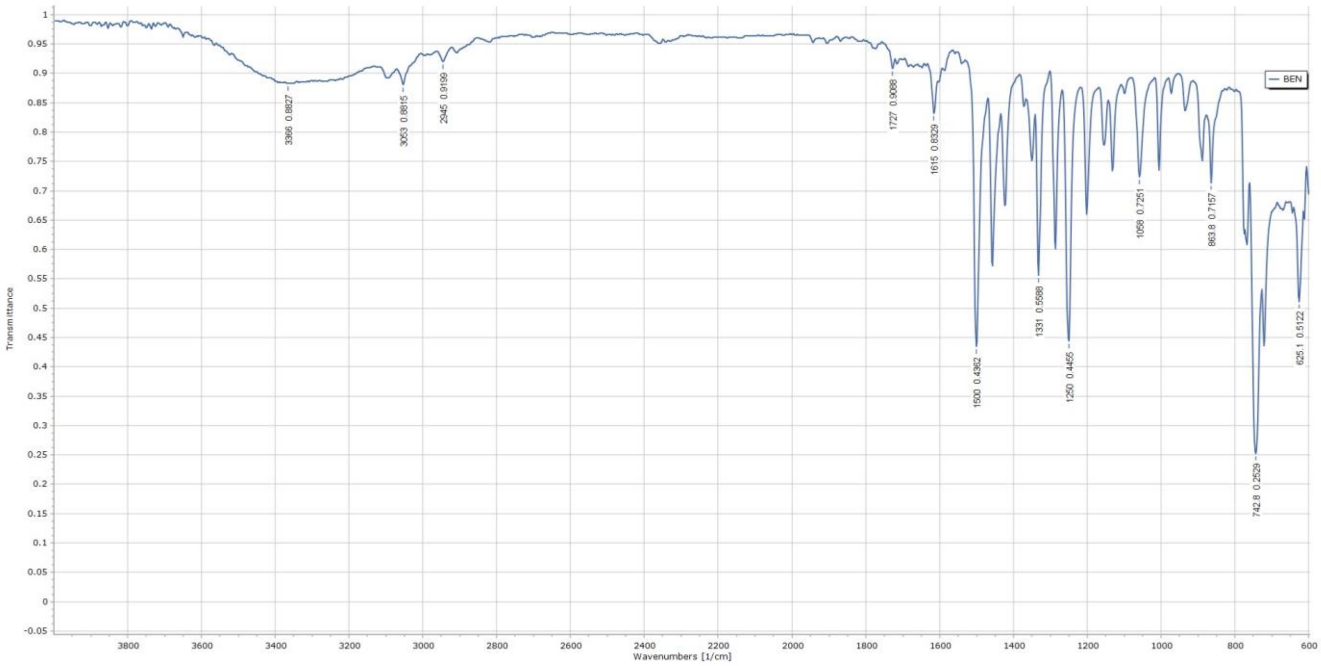

Figure S9. IR spectrum of *N*-methyl benzimidazole.

A

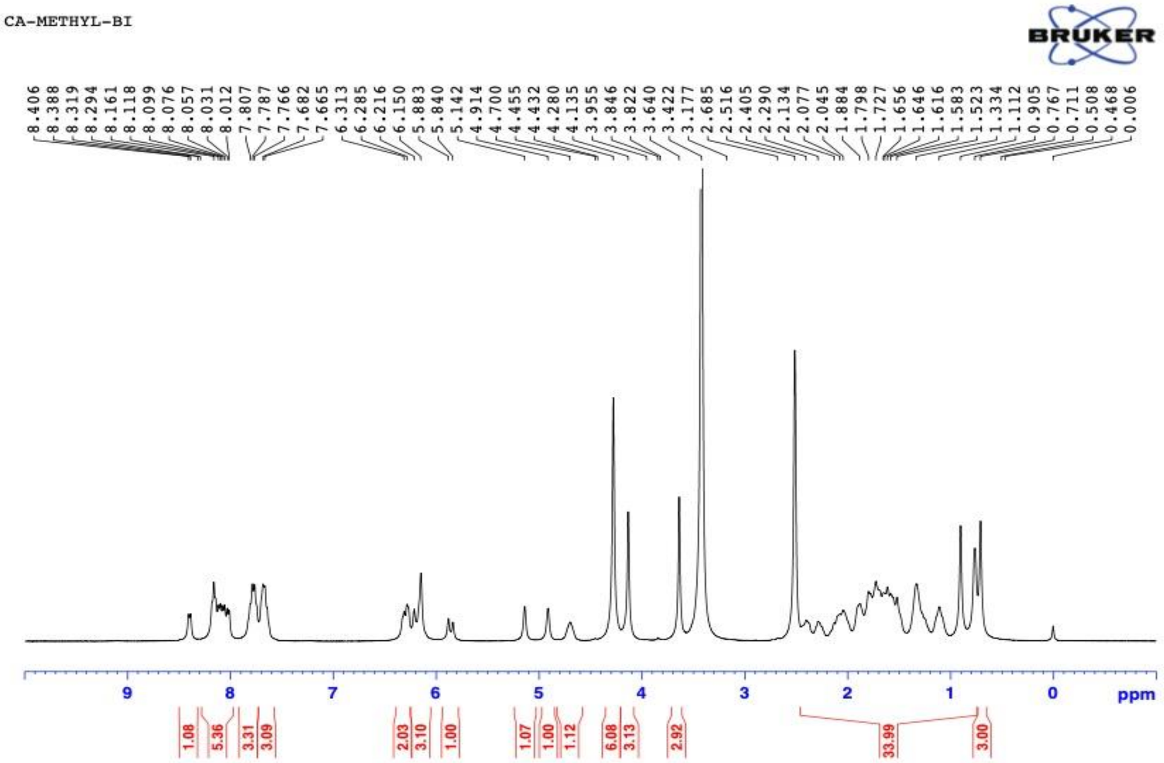

B

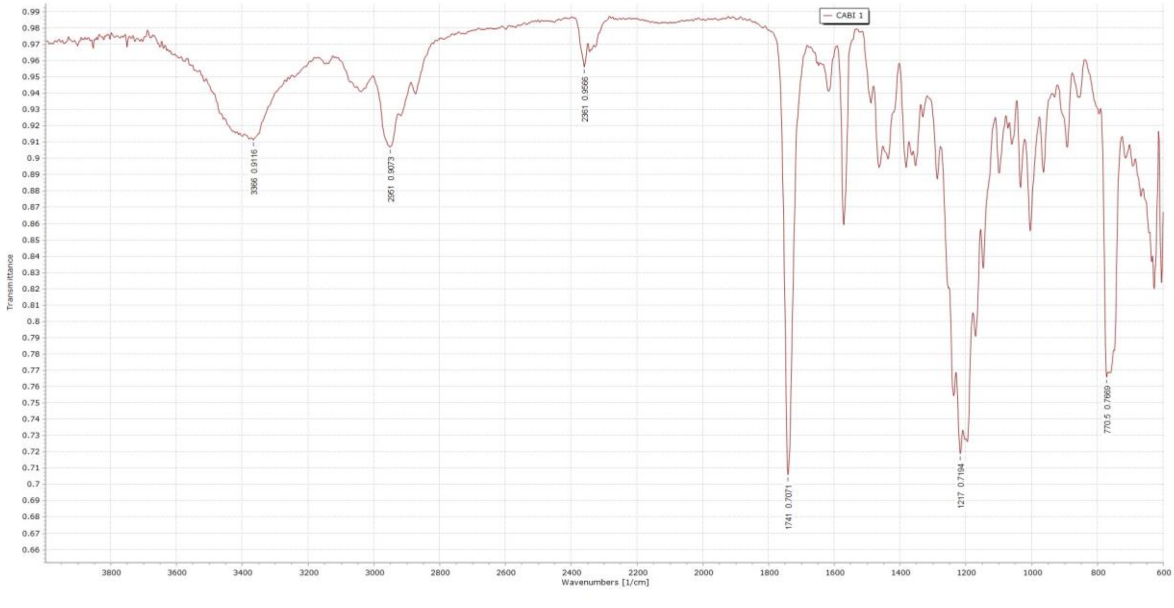

Figure S10. **(A**) ^{1}H NMR spectrum of CABI-**1**. **(B)** IR spectrum of CABI-**1**.

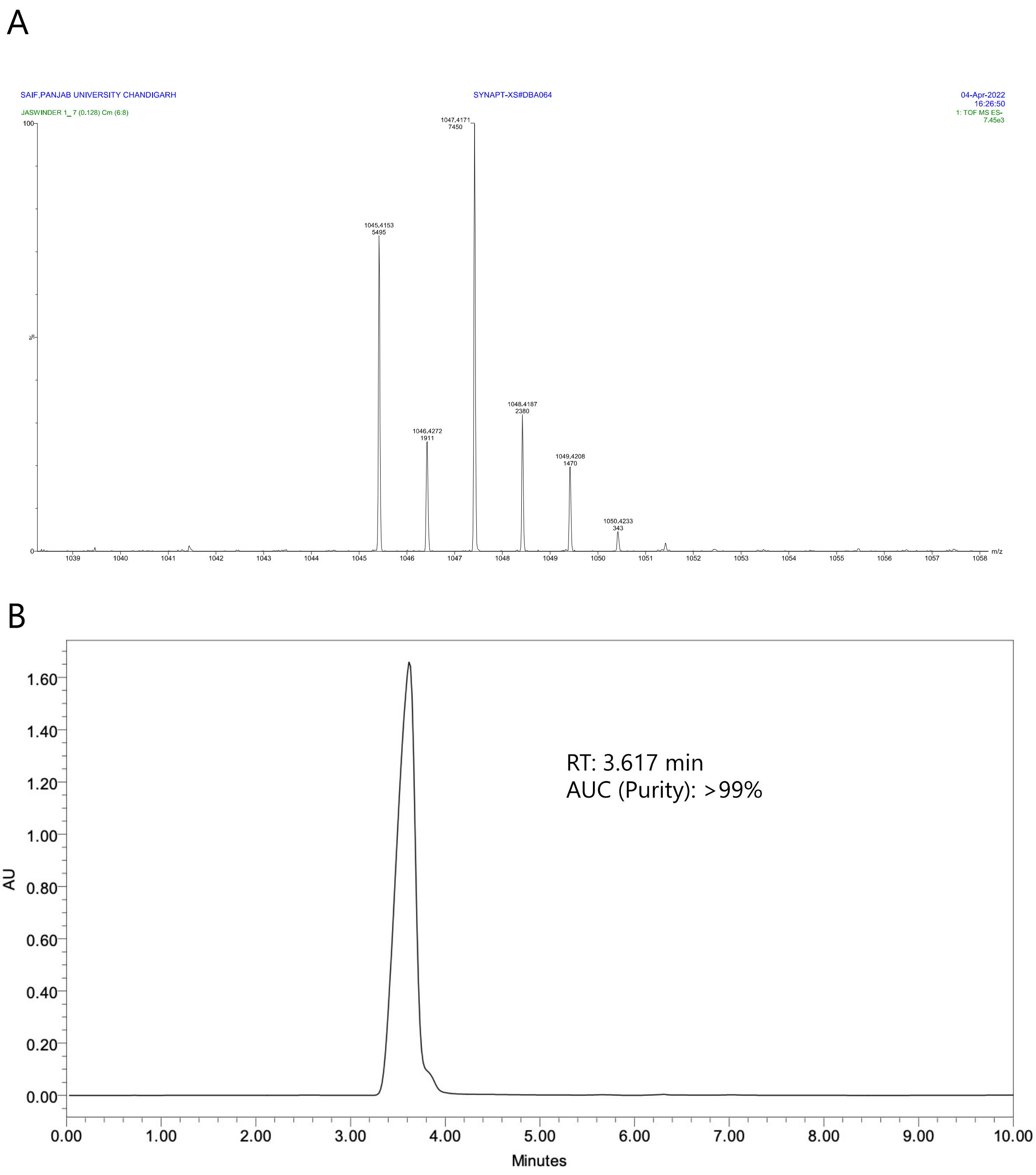

Figure S11. **(A)** Mass spectrum of CABI-**1**. **(B)** HPLC chromatograph of CABI-**1.**

A

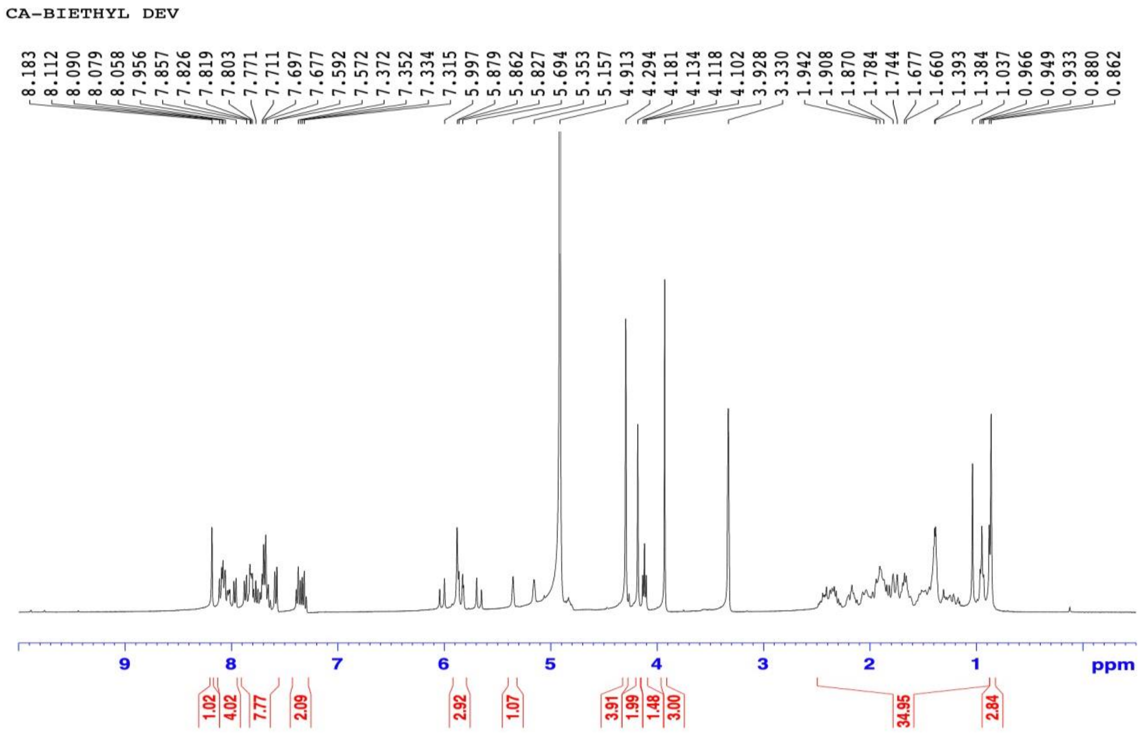

B

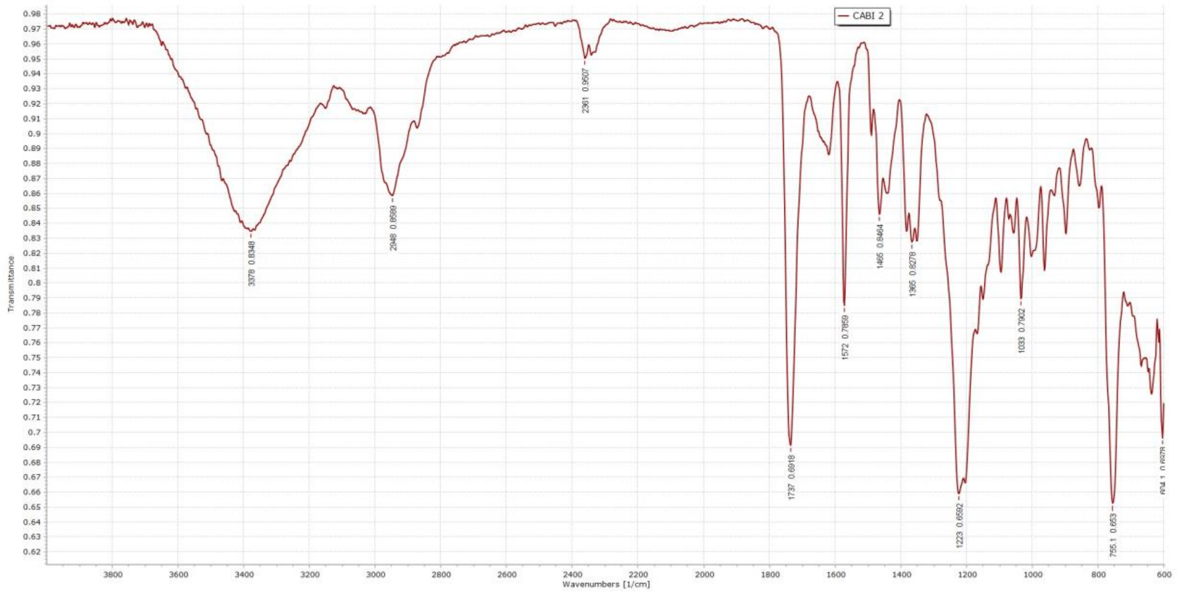

Figure S12. (**A**) ^{1}H NMR spectrum of CABI-**2**. **(B)** IR spectrum of CABI-**2**.

A

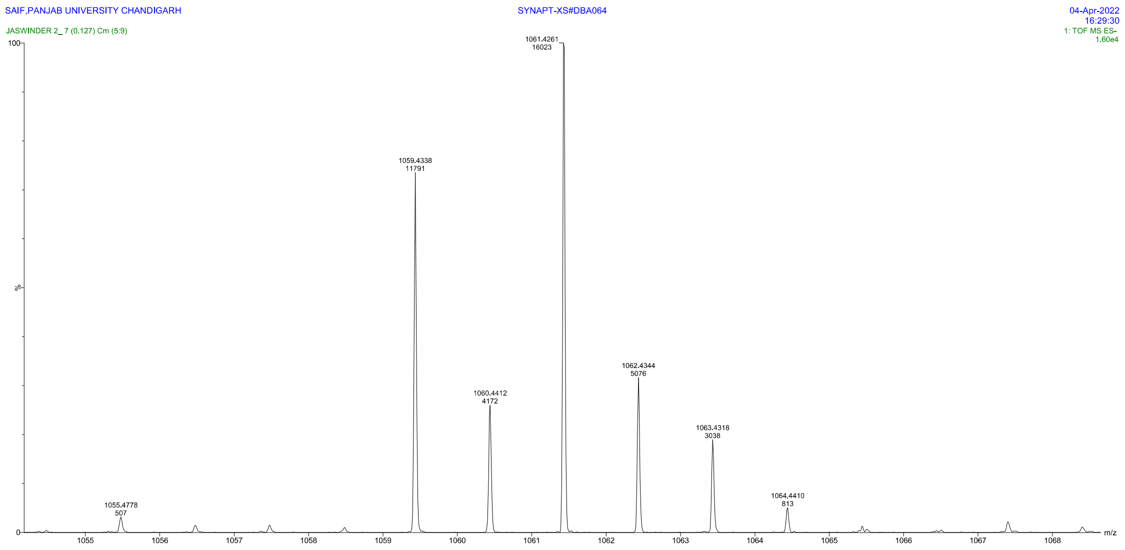

B

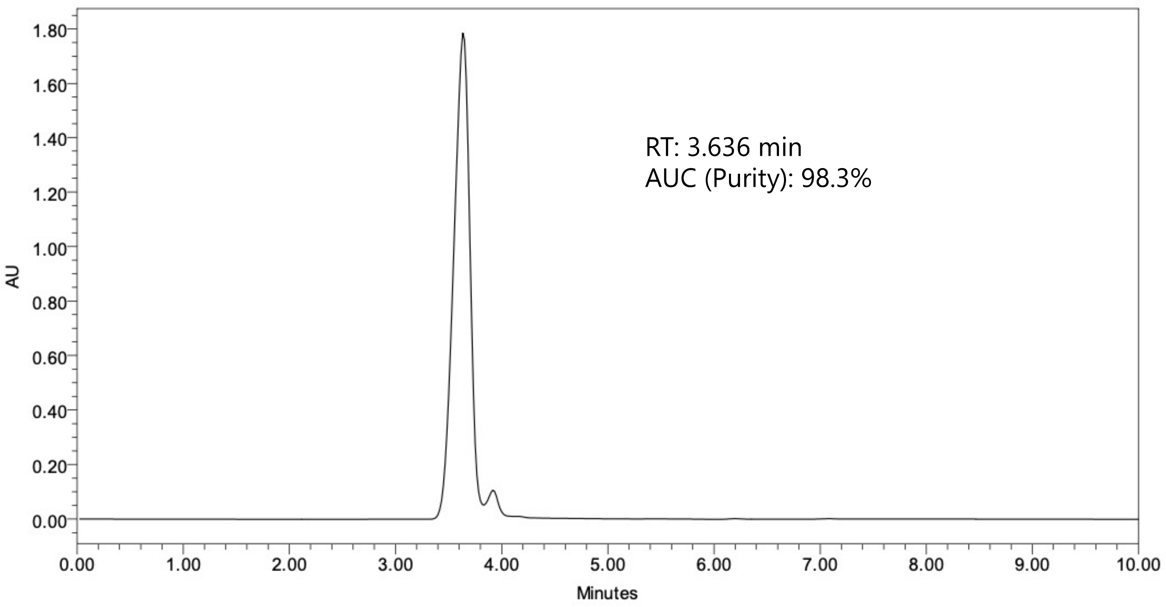

Figure S13. (**A**) Mass spectrum of CABI-**2**. **(B)**. IR spectrum of CABI-**2**.

A

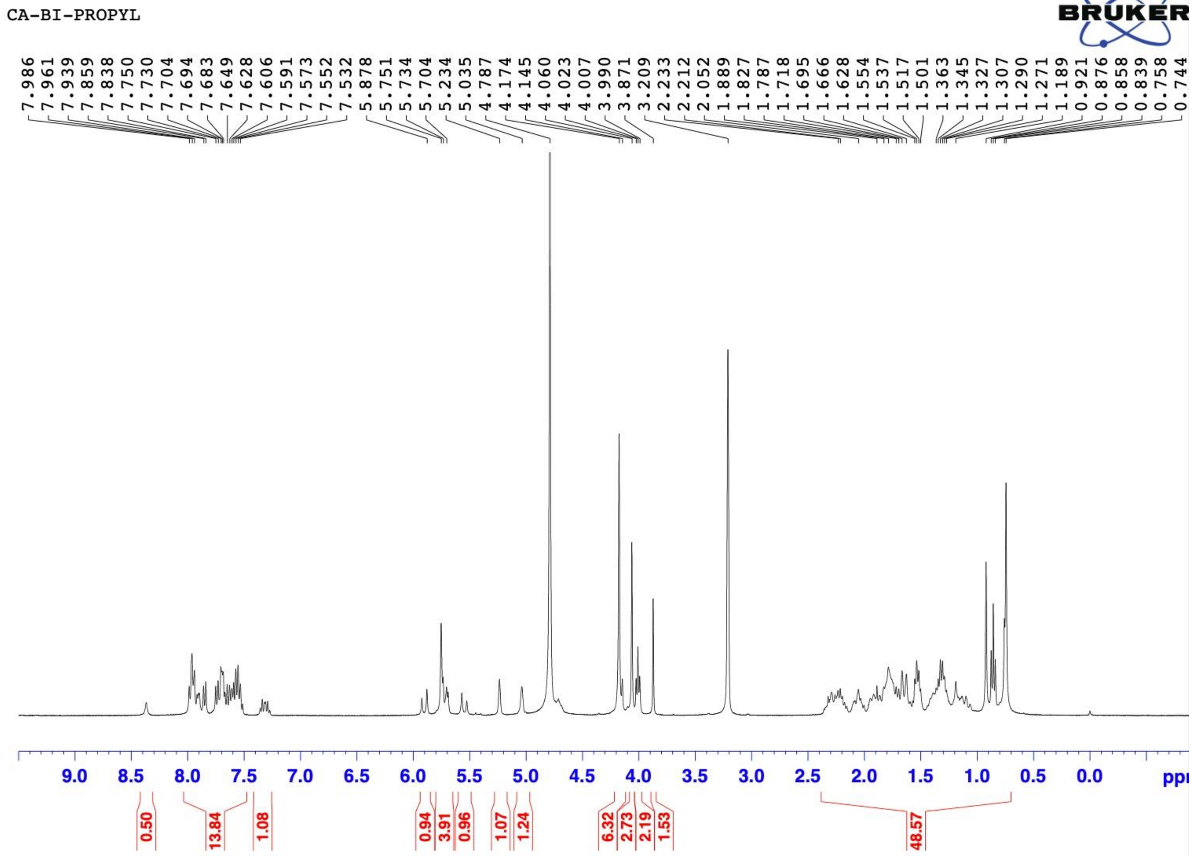

B

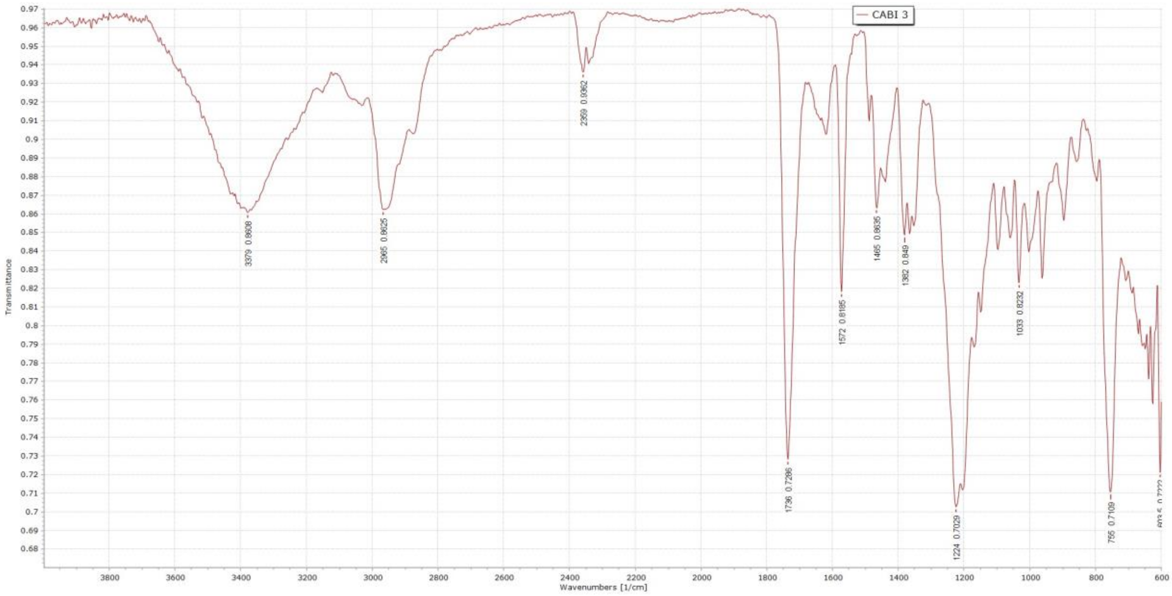

Figure S14. **(A)** ^{1}H NMR spectrum of CABI-**3**. **(B)**. IR spectrum of CABI-**3**.

A

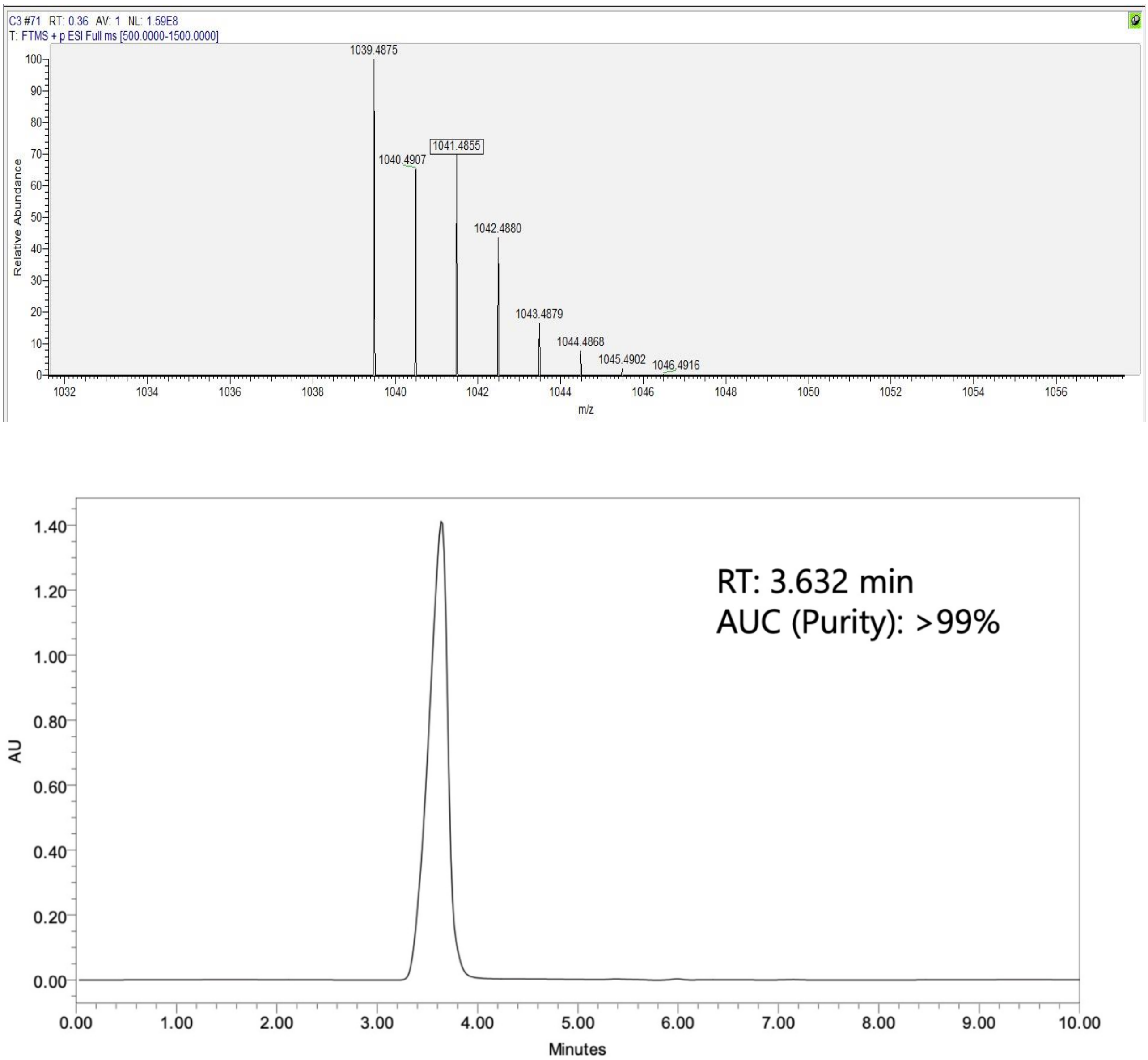

Figure S15. **(A)** Mass spectrum of CABI-**3**. **(B)** HPLC chromatograph of CABI-**3**.

A

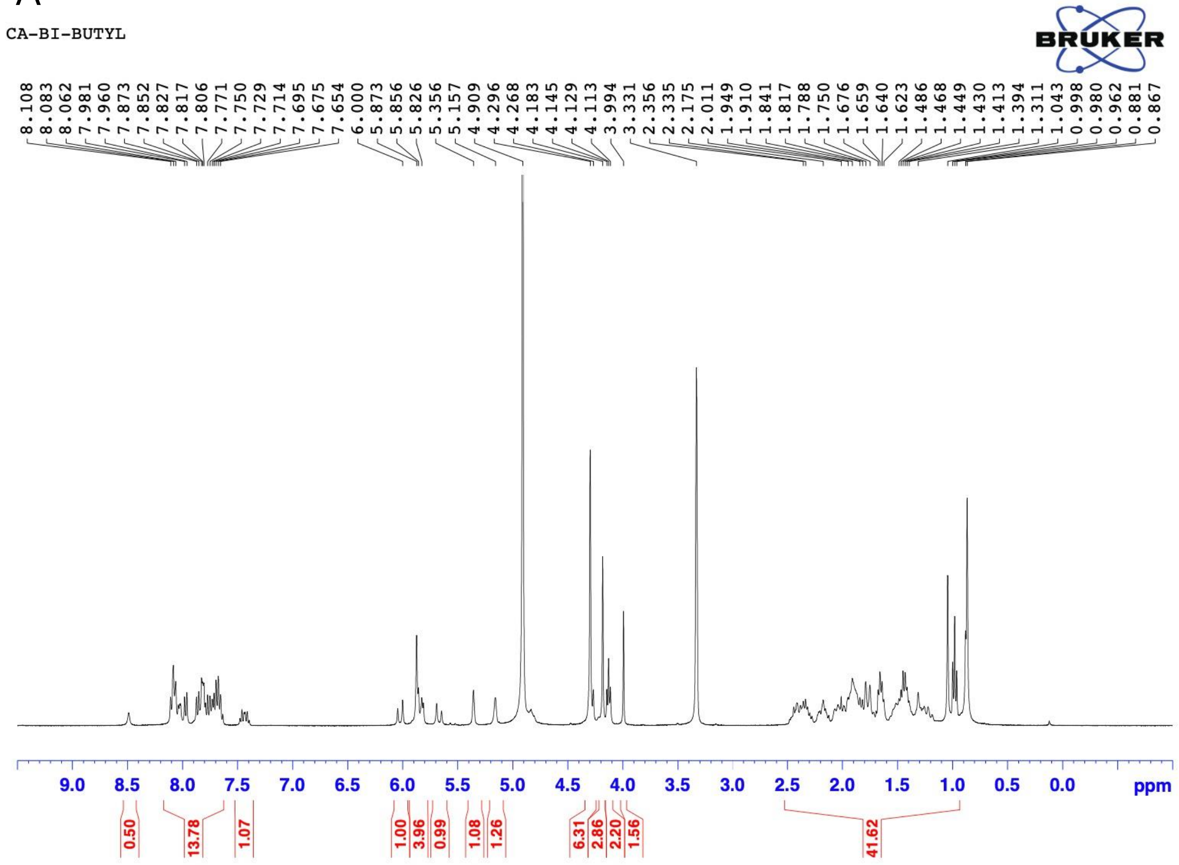

B

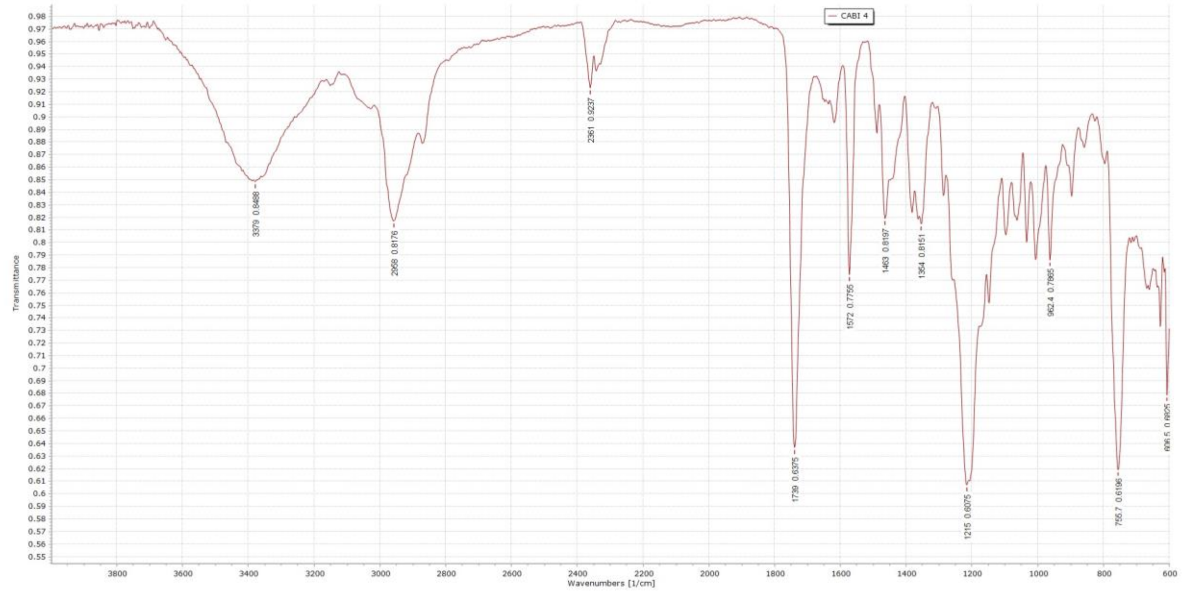

Figure S16. **(A)** ^{1}H NMR spectrum of CABI-**4**. **(B)** IR spectrum of CABI-**4**.

A

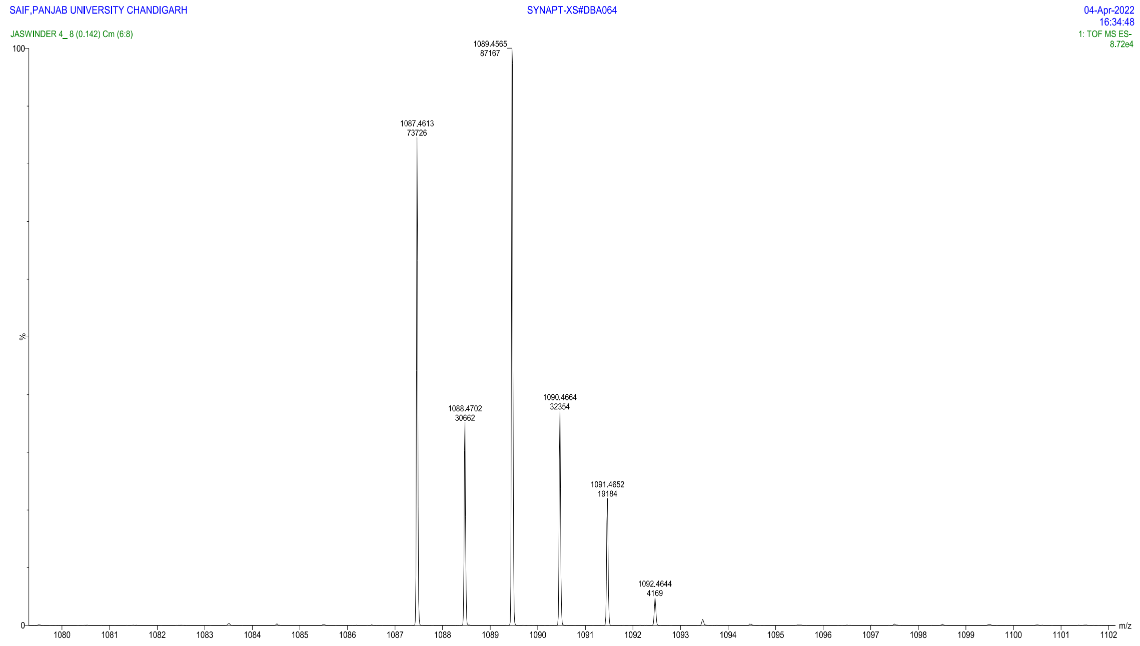

B

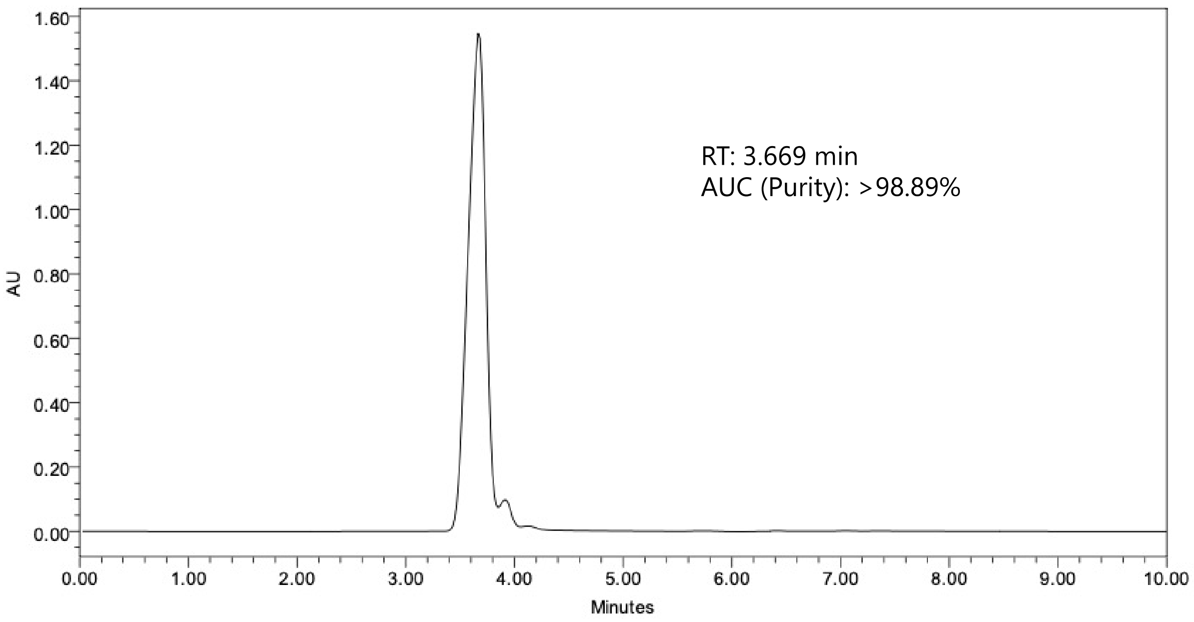

Figure S17. **(A)** Mass spectrum of CABI-**4**. **(B)** HPLC chromatograph of CABI-**4**.

A

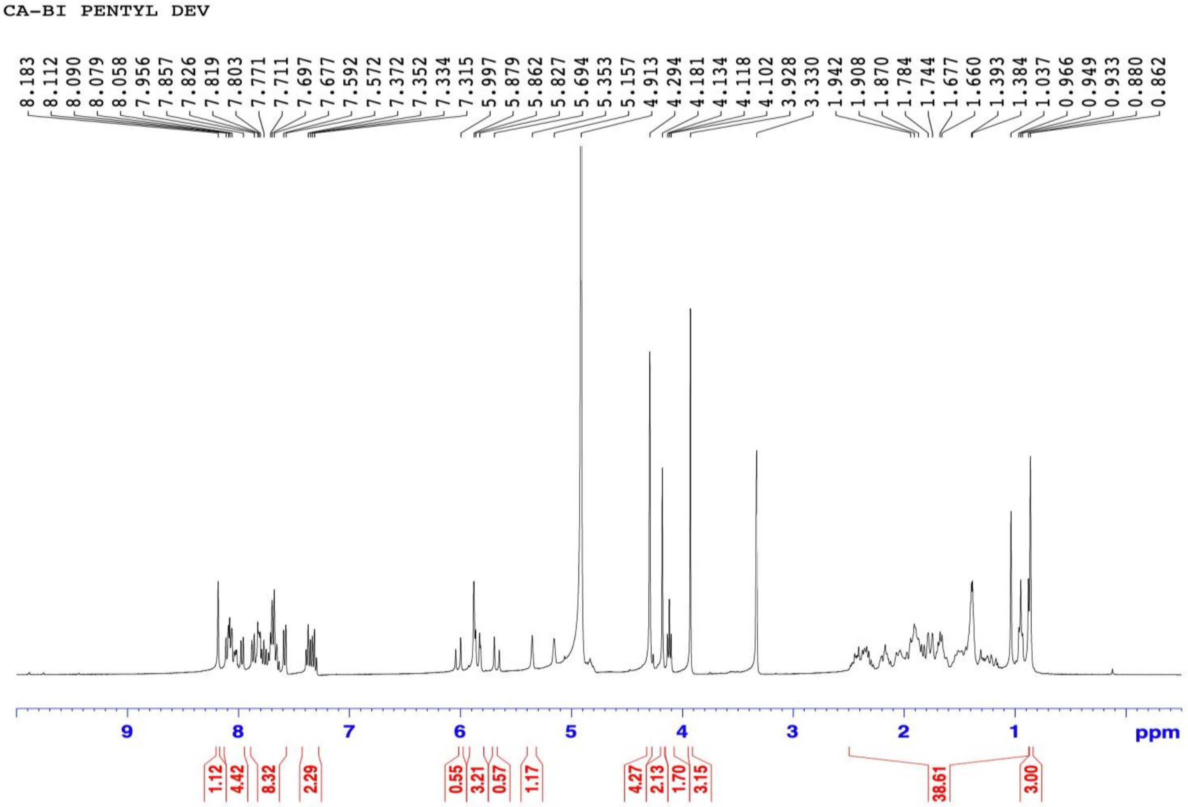

B

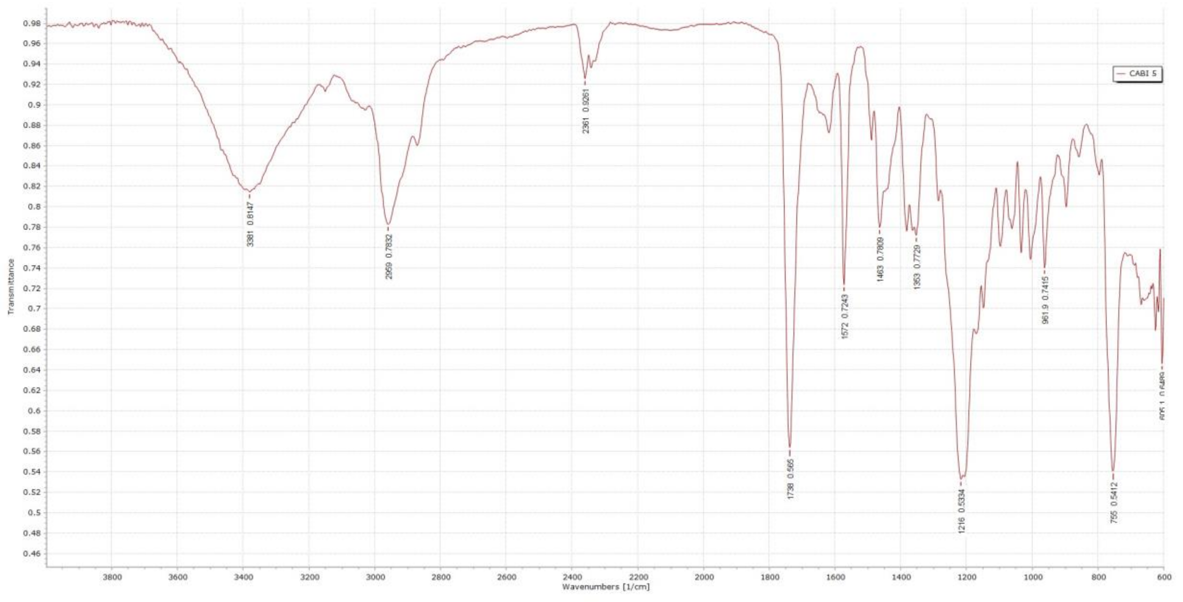

Figure S18. **(A)** ^{1}H NMR spectrum of CABI-**5**. **(B)** IR spectrum of CABI-**5**.

A

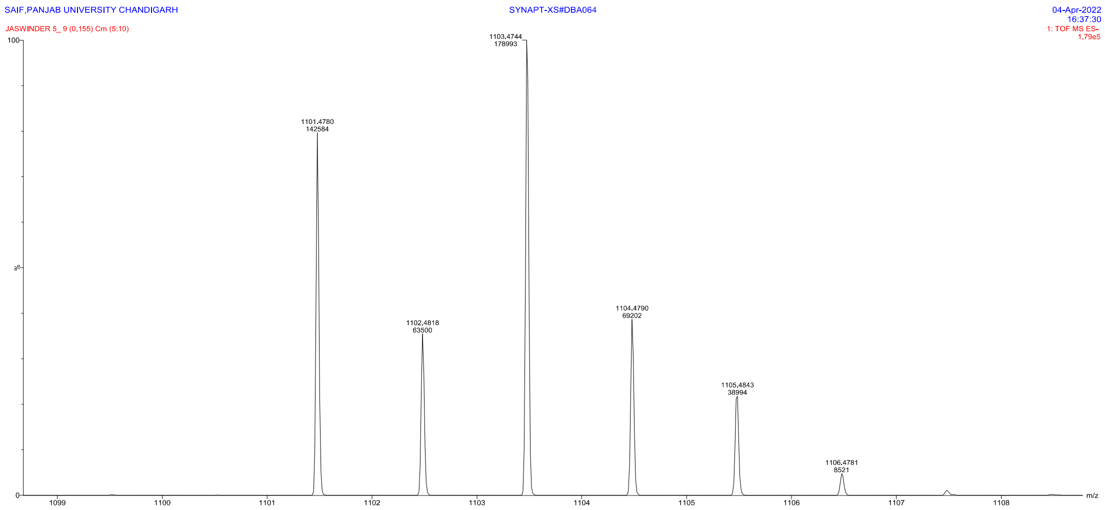

B

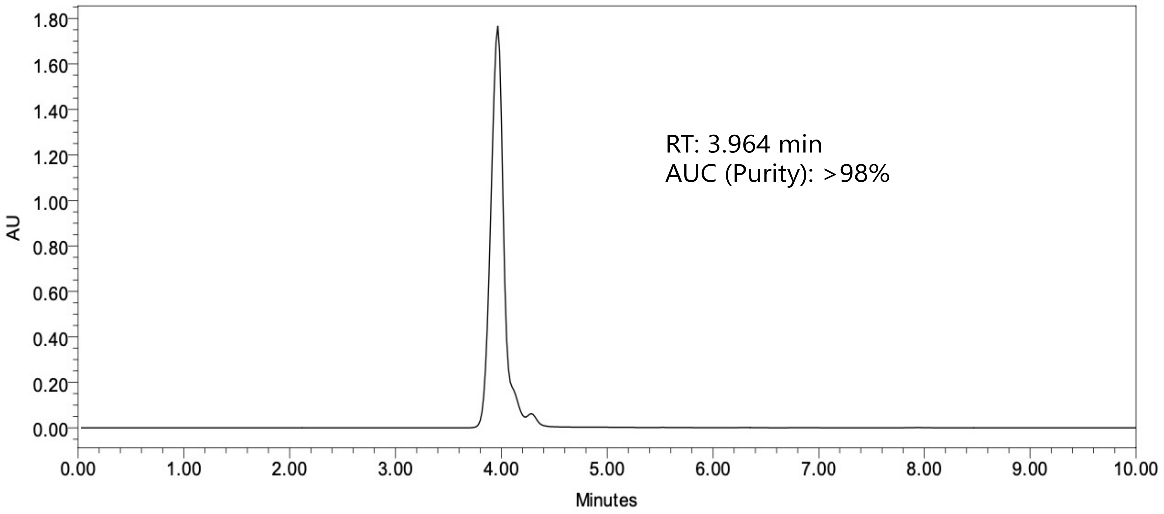

Figure S19. **(A)** Mass spectrum of CABI-**5**. **(B)** HPLC chromatograph of CABI-**5**.

A

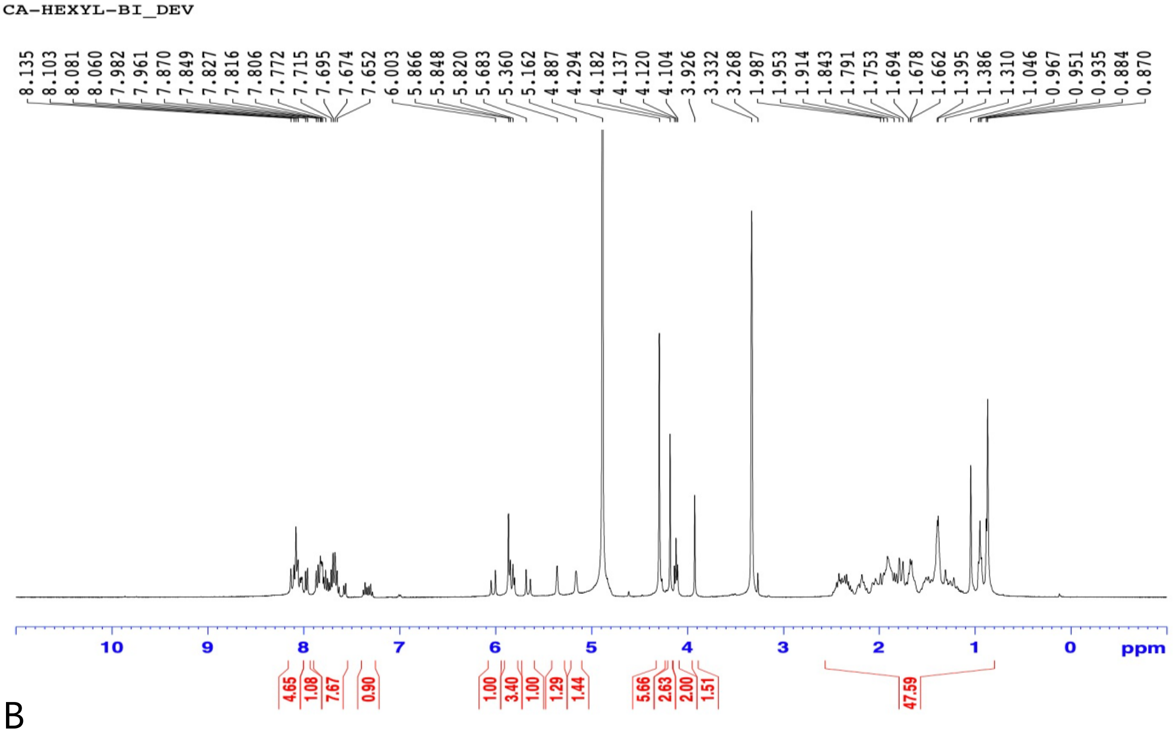

B

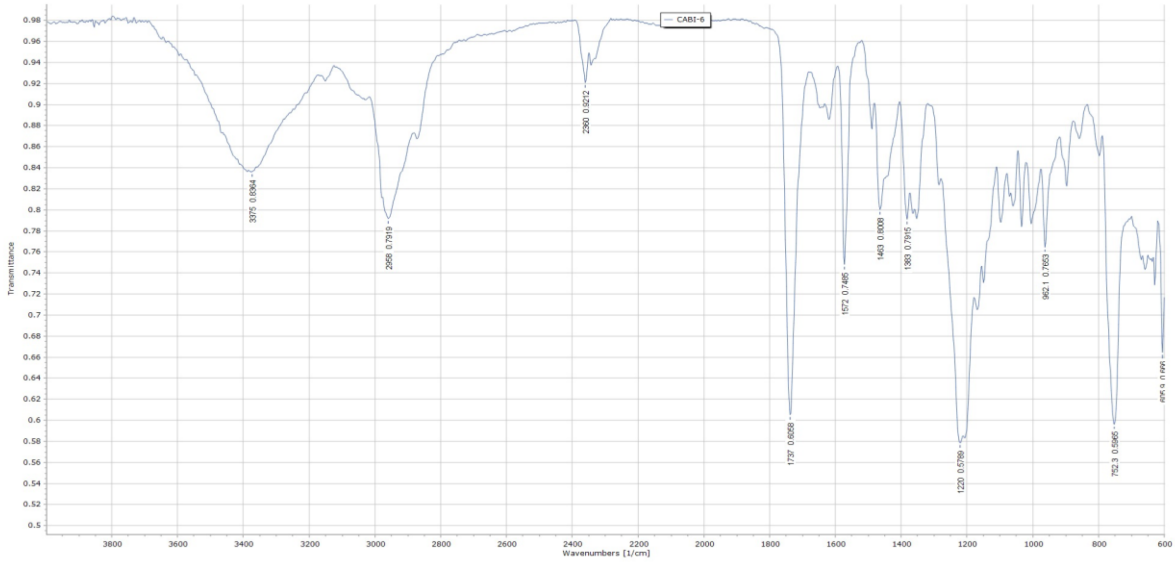

Figure S20. **(A)** ^{1}H NMR spectrum of CABI-**6**. **(B)** IR spectrum of CABI-**6**.

A

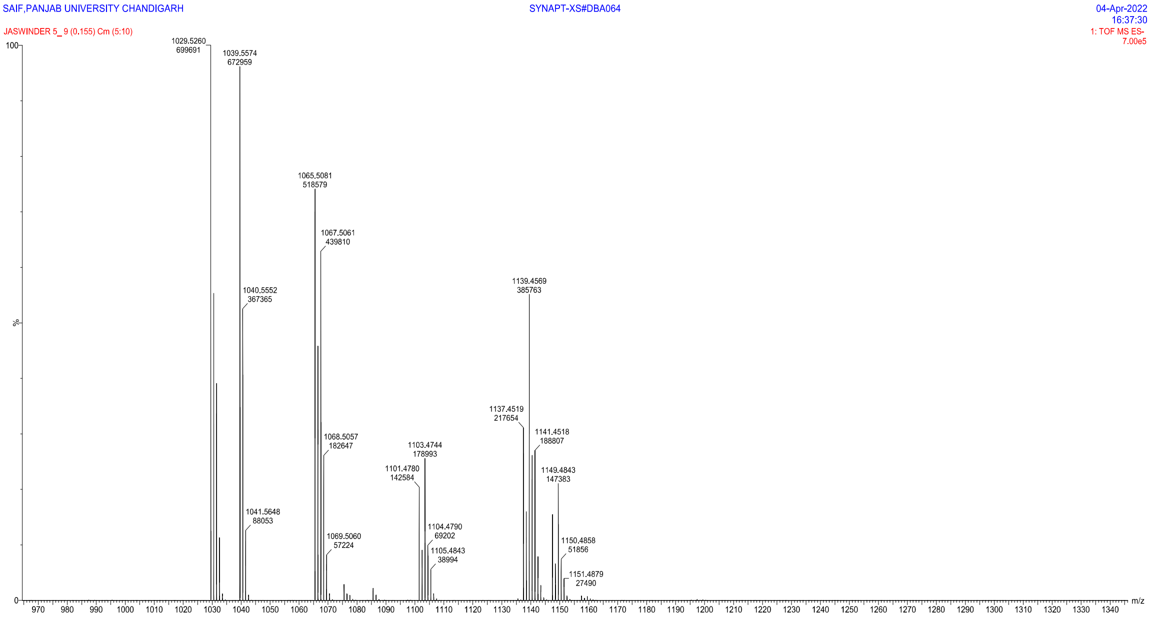

B

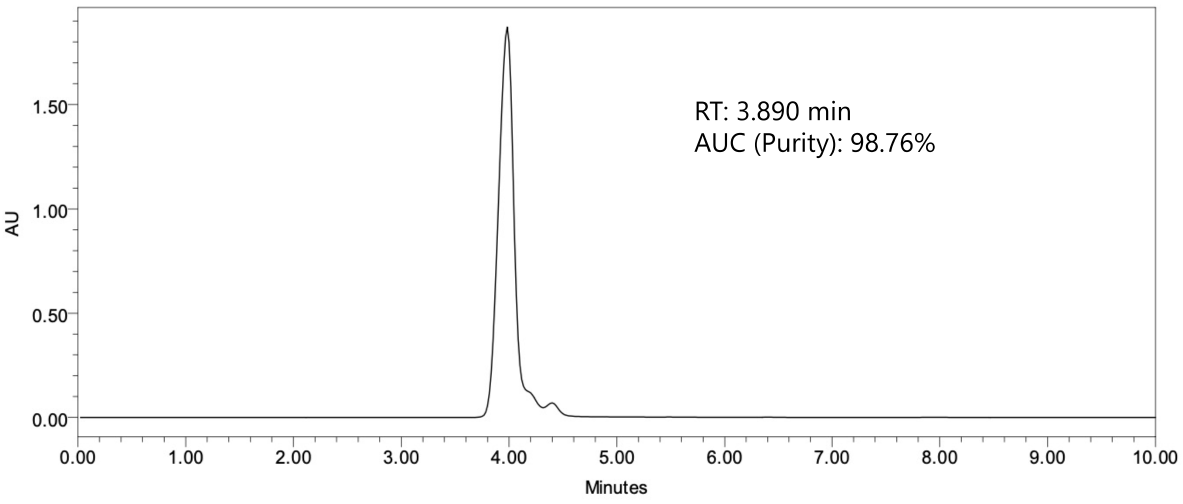

Figure S21. **(A)** Mass spectrum of CABI-**6**. **(B)** HPLC chromatograph of CABI-**6**.

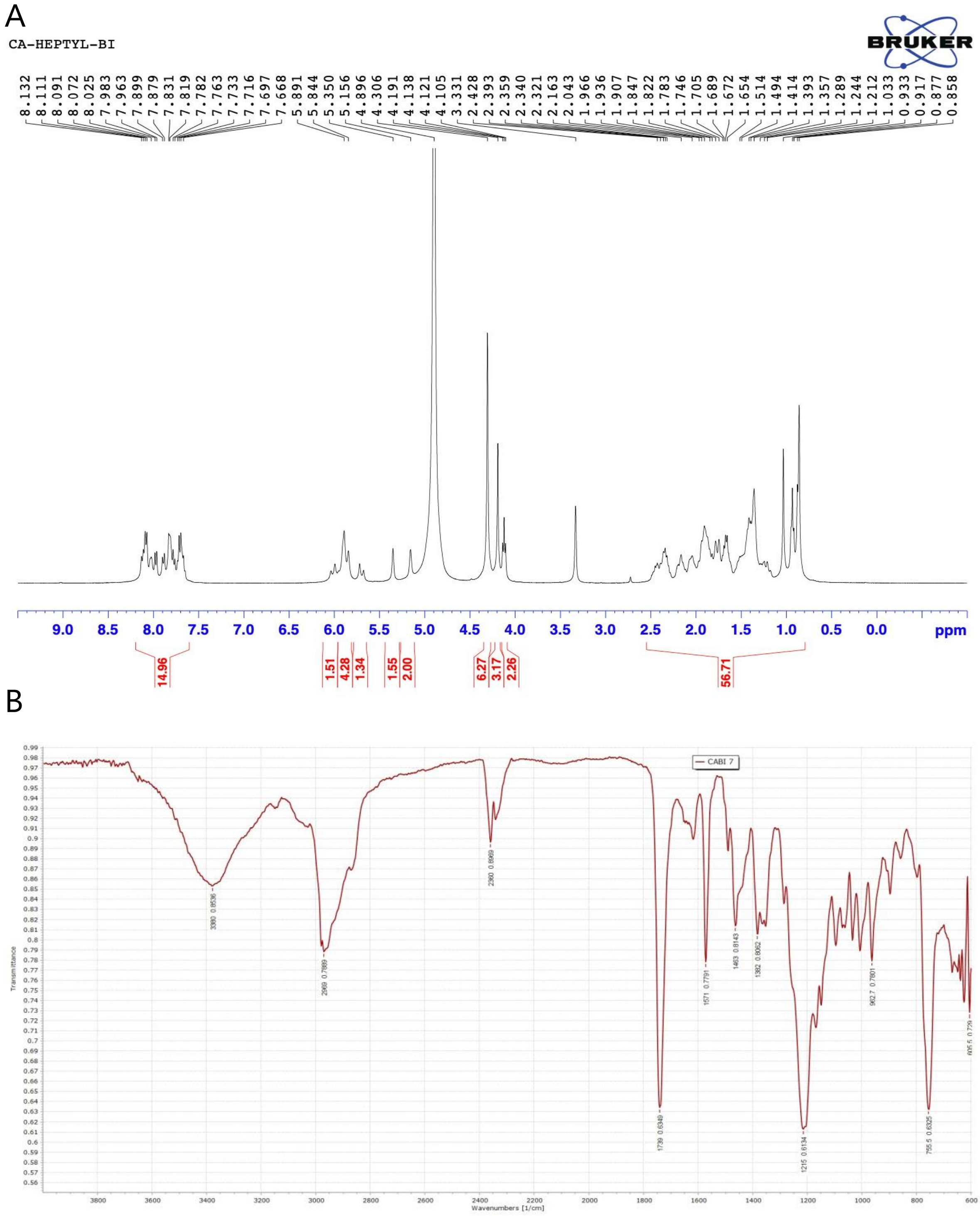

Figure S22. **(A)** ^{1}H NMR spectrum of CABI-**7**. **(B)** IR spectrum of CABI-**7**.

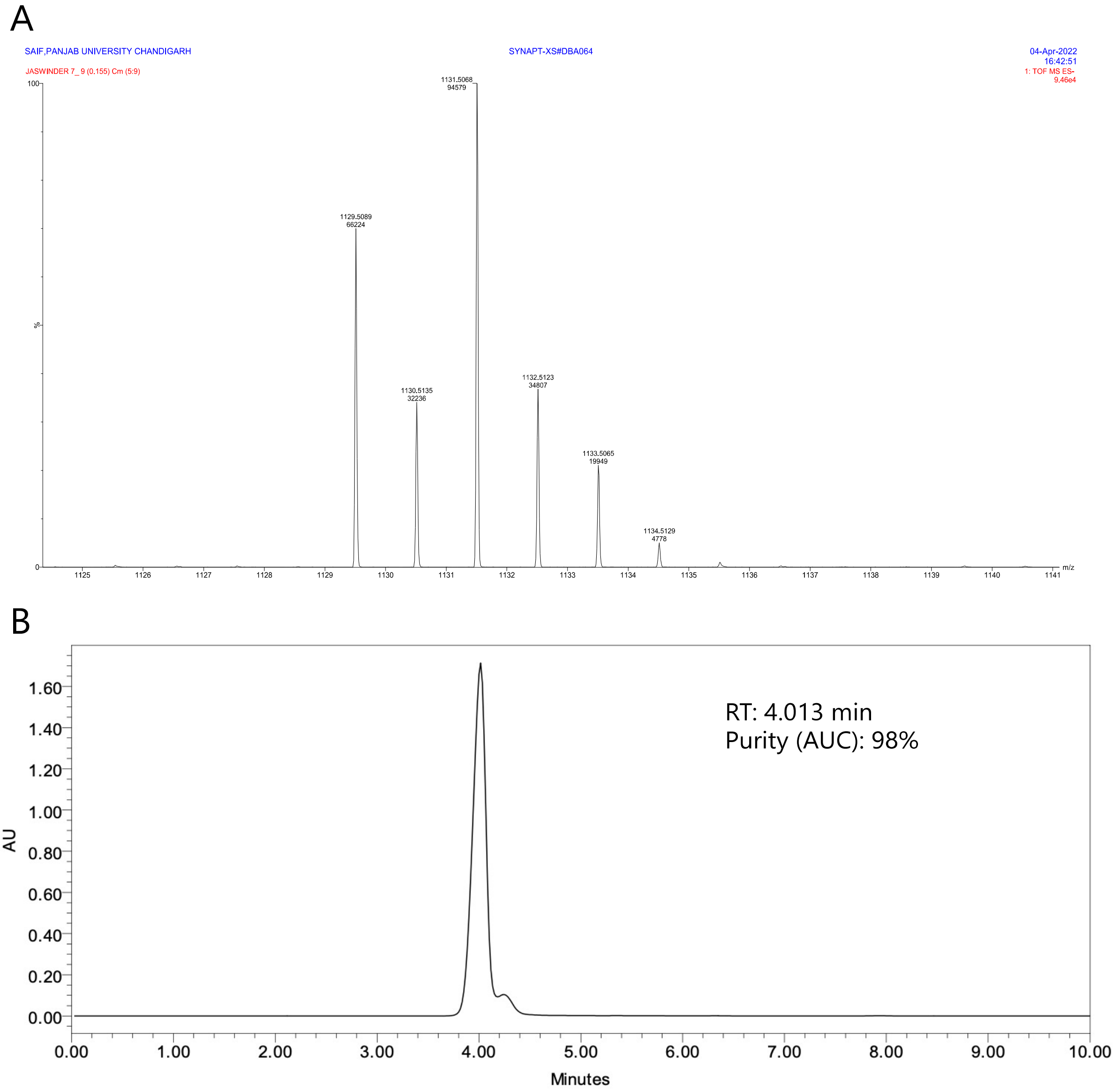

Figure S23. (A) Mass spectrum of CABI-**7**. **(B)** HPLC chromatograph of CABI-**7**.

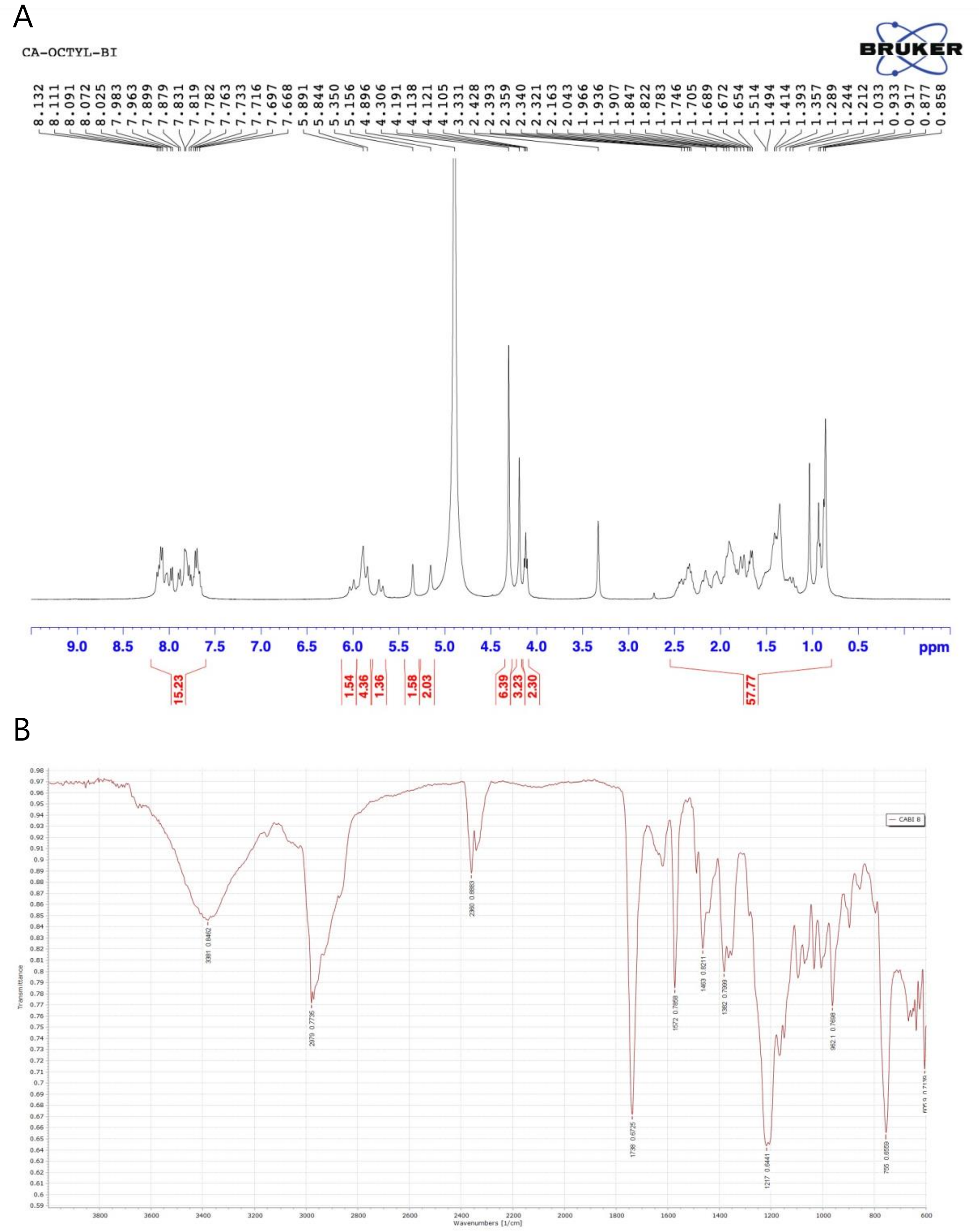

Figure S24. (A) ^{1}H NMR spectrum of CABI-**8**. **(B)** IR spectrum of CABI-**8**.

A

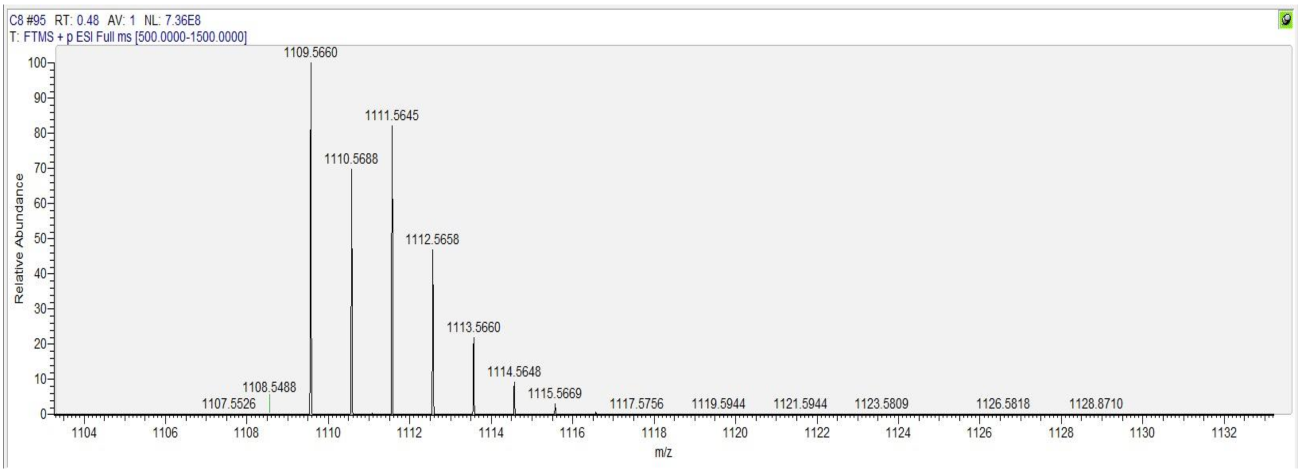

B

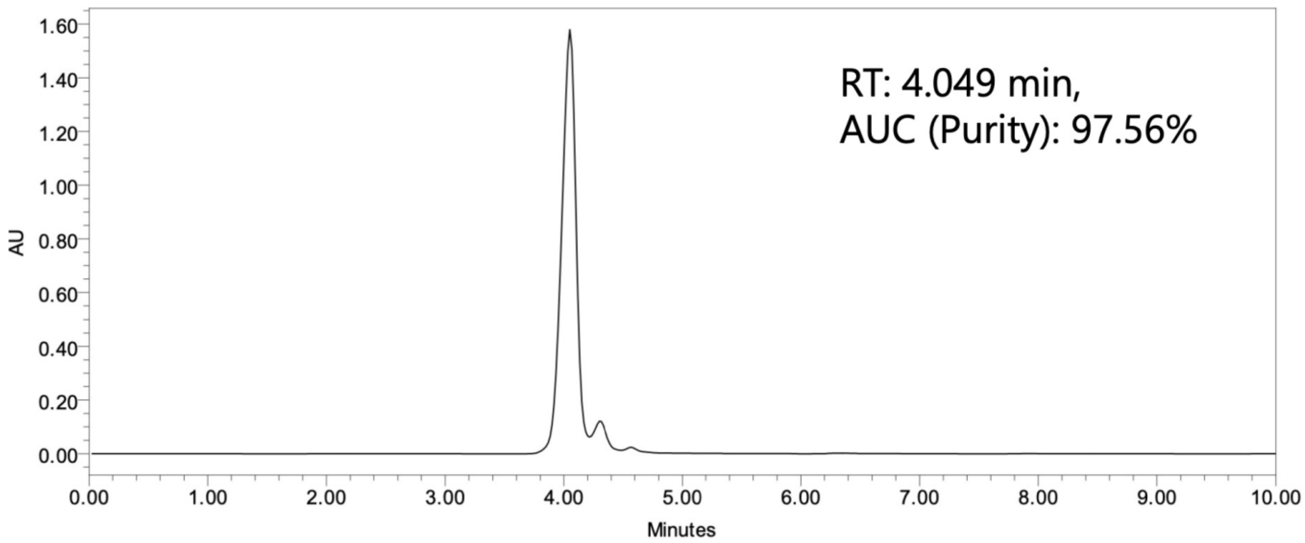

Figure S25. (A) Mass spectrum of CABI-**8**. **(B)** HPLC chromatograph of CABI-**8**.
